# Controllability in an islet specific regulatory network identifies the transcriptional factor NFATC4, which regulates Type 2 Diabetes associated genes

**DOI:** 10.1038/s41540-018-0057-0

**Published:** 2018-07-03

**Authors:** Amitabh Sharma, Arda Halu, Julius L. Decano, Megha Padi, Yang-Yu Liu, Rashmi B. Prasad, Joao Fadista, Marc Santolini, Jörg Menche, Scott T. Weiss, Marc Vidal, Edwin K. Silverman, Masanori Aikawa, Albert-László Barabási, Leif Groop, Joseph Loscalzo

**Affiliations:** 1 Channing Division of Network Medicine, Department of Medicine, Brigham and Women’s Hospital, Harvard Medical School, Boston, MA 02115 USA; 2 Center for Complex Network Research and Department of Physics, Northeastern University, Boston, MA 02115 USA; 3 Center for Cancer Systems Biology (CCSB), Dana-Farber Cancer Institute, Boston, MA 02215 USA; 4 Center for Interdisciplinary Cardiovascular Sciences, Cardiovascular Division, Brigham and Women’s Hospital, Harvard Medical School, Boston, MA 02215 USA; 5 Department of Molecular and Cellular Biology, University of Arizona, Tucson, AZ 85721 USA; 6 Lund University Diabetes Center, Department of Clinical Sciences, Diabetes & Endocrinology, Skåne University Hospital Malmö, Lund University, Malmö, 20502 Sweden; 7 CeMM Research Center for Molecular Medicine of the Austrian Academy of Sciences, Vienna, 1090 Austria; 8 Department of Genetics, Harvard Medical School, Boston, MA 02115 USA; 9 Center for Network Science, Central European University, Nador u. 9, 1051 Budapest, Hungary; 10 Department of Clinical Sciences, Islet cell physiology, Skåne University Hospital Malmö, Lund University, Malmö, 20502 Sweden; 11 Department of Medicine, Brigham and Women’s Hospital, Harvard Medical School, Boston, MA 02115 USA

## Abstract

Probing the dynamic control features of biological networks represents a new frontier in capturing the dysregulated pathways in complex diseases. Here, using patient samples obtained from a pancreatic islet transplantation program, we constructed a tissue-specific gene regulatory network and used the control centrality (Cc) concept to identify the high control centrality (HiCc) pathways, which might serve as key pathobiological pathways for Type 2 Diabetes (T2D). We found that HiCc pathway genes were significantly enriched with modest GWAS *p*-values in the DIAbetes Genetics Replication And Meta-analysis (DIAGRAM) study. We identified variants regulating gene expression (expression quantitative loci, eQTL) of HiCc pathway genes in islet samples. These eQTL genes showed higher levels of differential expression compared to non-eQTL genes in low, medium, and high glucose concentrations in rat islets. Among genes with highly significant eQTL evidence, NFATC4 belonged to four HiCc pathways. We asked if the expressions of T2D-associated candidate genes from GWAS and literature are regulated by Nfatc4 in rat islets. Extensive in vitro silencing of Nfatc4 in rat islet cells displayed reduced expression of 16, and increased expression of four putative downstream T2D genes. Overall, our approach uncovers the mechanistic connection of NFATC4 with downstream targets including a previously unknown one, TCF7L2, and establishes the HiCc pathways’ relationship to T2D.

## Network medicine: Identifying the controlling pathways of Type 2 Diabetes

Genes that structurally control regulatory networks uncover the pathways driving complex diseases. Amitabh Sharma of Brigham and Women’s Hospital and his colleagues constructed a tissue-specific gene regulatory network derived from human pancreatic islets and determined the genes that control the network, using the concept of “control centrality.” Pathways with high control centrality were significantly more associated with Type 2 Diabetes (T2D) than pathways with lower control centrality, and harbored loci that significantly affected gene expression (eQTLs) related to glucose levels. In vitro knockdown of NFATC4, jointly identified by control centrality and eQTL analysis, verified its downstream influence on genes known to be associated with T2D. Controllability in biological networks may help us pinpoint influential pathways as well as key regulators of many other complex diseases.

## Introduction

The pathobiological changes leading to a complex disease are most likely to be influenced by the disease genes that perturb the underlying biological networks in specific tissue types. Recent evidence suggests that these perturbations are not scattered randomly in the interactome; instead, they are localized in specific neighborhoods, or ‘disease modules’.^1,2^ In order to identify this disease-specific interactome neighborhood, we previously integrated human islet gene expression data, genetics, and protein interaction data to build a localized map of genes associated with islet cell dysfunction in Type 2 Diabetes (T2D).^3^ Recently, we identified an asthma disease module by a connectivity-based model and validated it for functional and pathophysiological relevance to the disease.^2^ Several tools based on the ‘guilt-by-association principle’ predict potential candidate genes using networks.^4–6^ Furthermore, inference tools such as ANAT identify the highest-confidence paths between pairs of proteins by viewing the local neighborhood of a set of proteins.^7^ Other methods such as HotNet2 use the heat diffusion process to analyze a gene’s mutation score and its local topology together to find the subnetworks in cancer.^8^ Similarly, the NetQTL approach combines eQTL and network flow to identify genes and dysregulated pathways.^9^

Despite extensive interest in using topological features to interpret the biological networks in human disease, an important aspect that has been largely overlooked so far is the *controllability* of these subcellular networks. In general, controllability can be achieved by changing the state of a small set of driver nodes that govern the dynamics of the entire network.^10^ Liu et al.^11^ proposed an analytical framework to identify the minimum set of driver nodes (MDS) of any complex network, whose time-dependent control can guide the whole network to any desired final state. They found that driver nodes tend to avoid hubs, i.e., highly connected nodes. Furthermore, Milenkovic et al.^12^ suggested the notion of domination and found dominating sets (DSs) in the undirected protein interaction network. Wuchty identified the minimum dominating sets (MDSets) that play a role in the control of the underlying protein interaction network.^9^ It was observed that MDSet proteins were enriched with cancer-related and virus-targeted genes.^13^ We recently showed that the application of network controllability analysis helps in identifying new disease genes and drug targets.^14^

**Fig. 1:**
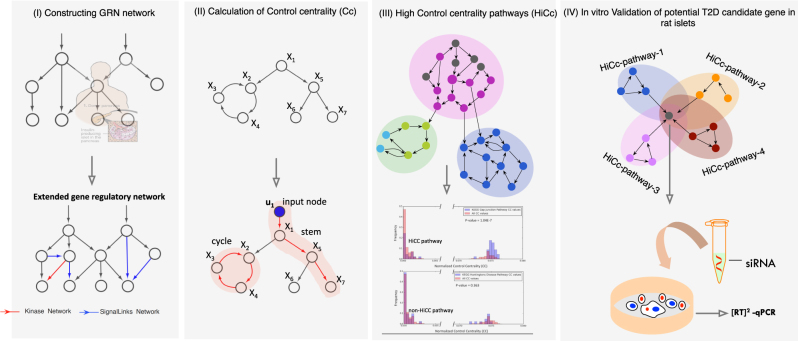
Overview of the approach to identify the key pathways in T2D using control centrality approach. **a** Gene expression data: Pancreatic islets from cadaver donors (54 nondiabetic and 9 diabetic) were used to construct the gene regulatory network (GRN) and extended by adding kinase and signaling links. The largest connected component of the extended GRN (EGRN) consists of *N* = 3084 genes and *M* = 7935 edges. **b** The *c*ontrol centrality measure is used to quantify the relative importance of each gene in EGRN relative to T2D. **c** High control centrality (HiCc) pathways are found by comparing the control centrality distribution of genes within the pathway vs the control centrality distribution of all other genes in EGRN. Pathways with a significantly higher control centrality distribution compared to the background are deemed HiCc pathways. For example, the Gap junction pathway emerges as a HiCc pathway, whereas the Huntington’s Disease pathway is found to be a non-HiCc pathway. **d** In vitro silencing experiments are performed on genes implicated in a large number of HiCc pathways, such as NFATC4, to discover novel mechanistic connections with known T2D genes

Progress towards a robust network-based controllability approach will ultimately lead to the identification of potential key regulatory nodes that govern network function in health and disease. As a first step in this direction, here, we asked whether the set of genes that are predicted to control a biological directed network would affect the functional pathophenotype. To assess the controllability of the network, we used the control centrality (Cc) measure,^15^ which quantifies the ability of a single node to control an entire directed weighted network (see 'Methods'). Our disease of interest, T2D, is a complex disease and therefore has the potential to lend itself to this approach where controllability in a regulatory network specific to it might reveal new knowledge about the disease. T2D is characterized by insufficient insulin secretion from the β-cells of islets in the pancreas.^3^ We hypothesize that the high control centrality (HiCc) pathways, representing specific gene sets in a T2D-regulatory network in human pancreatic islets, might control other downstream pathways involved in disease manifestation (see Fig. 1). To test our hypothesis we construct a pancreatic islet-specific EGRN, and use Cc to identify HiCc pathways in the KEGG database. We validate the disease relevance of these HiCc pathways using T2D-specific -omics data. Next, we test whether the SNPs located in non-coding regions of HiCc pathway genes in islet samples would influence gene expression (eQTL). Finally, we perform extensive in vitro silencing experiments on NFATC4, an eQTL-implicated gene found in four HiCc pathways, and probe whether T2D-associated genes from GWAS and literature are downstream targets of NFATC4. Overall, our study provides a unique framework for integrating control principles towards distinguishing pathways and genes that are likely to contribute to T2D pathogenesis.

## Results

### Extended gene-regulatory network (EGRN) from human islet cells

**Fig. 2:**
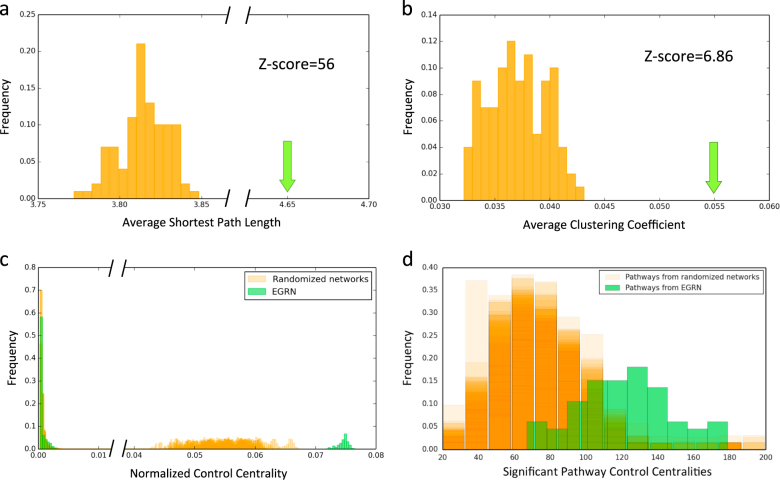
Topological and control centrality-related properties of EGRN. **a** The average shortest path length of the EGRN is 4.65, which is significantly higher that those of randomized networks (shown in orange) with a *z*-score of 56. **b** The average clustering coefficient of the EGRN is 0.055, which is significantly higher that those of randomized networks (shown in orange) with a *z*-score of 6.86. **c** The normalized control centrality distribution of the EGRN (shown in green) is significantly higher than those of randomized networks (shown in orange). **d** The control centralities of the HiCc pathways derived from the EGRN (shown in green) are significantly higher than those of the HiCc pathways derived from randomized networks

We start by building a gene-regulatory network (GRN) using gene expression data from pancreatic islet samples of diabetic and non-diabetic cadaver donors obtained through the Nordic Islet Transplantation Programme (http://www.nordicislets.org). The GRN consists of differentially expressed genes in diabetic and high glycated hemoglobin (HbA1c) donors, highly varying genes in all donor islets, and established T2D genes from genome-wide association studies (GWAS). Directed edges in the GRN are inferred using a combination of mutual information and prior knowledge from the TRANSFAC database (http://www.gene-regulation.com/; see 'Methods' for further details). The GRN specific to islet cells consists of 896 genes with 1164 links between them. We next include most of the signaling events by extending the GRN with the addition of kinase and signaling pathways (see 'Methods' for further details on the construction of the networks). The largest connected component (LCC) of the EGRN with kinase-substrate and signaling links consists of *N* = 3084 genes and *M* = 7935 edges. The average number of neighbors in the network is 5.14. Compared to randomized networks constructed using degree-preserving randomization, the EGRN has a significantly higher average shortest path length <*l*> = 4.65 (*z*-score = 56) (Fig. 2a) and a significantly higher clustering coefficient *C* = 0.055 (*z*-score = 6.86) (Fig. 2b). Interestingly, irrespective of the difference in network size and the degree distributions of GRN and EGRN (Supplementary Figure 1a), their normalized Cc (c_c_ = C_c_/*N*) distributions do not differ significantly (Supplementary Figure 1b). This indicates the robustness of the Cc measure to changes in the size of the network. Moreover, EGRN’s normalized Cc is significantly higher compared to randomized networks. The *p*-values between randomized networks and EGRN using Mann–Whitney *U* (100 *p*-values) are between 2.77e-15 and 9.76e-12 (Fig. 2c). This suggests that the overall structure of the EGRN is more controllable than its randomized counterparts.

### Identifying the HiCc pathways in the EGRN

A complex disease such as T2D is likely to be the result of multiple gene perturbations within pathways in a biological network, where changes in one pathway might trigger alterations in other downstream pathways. Hence, identification of ‘key driver pathways’ in the islet-specific EGRN should give us insights about the molecular processes responsible for the disease. Here, we compared the Cc distribution of the genes in each pathway in the KEGG database with the Cc distribution of all other genes in the EGRN, and observed 66 significant HiCc pathways with a *p*-value < 0.05 (Mann–Whitney *U* test) (Supplementary Table 2) (for details see 'Methods'). Overall, the genes representing T2D pathways in the EGRN had higher C_c_ values compared to the random distribution (Supplementary Figure 2), indicating that Cc is able to capture the important pathways associated with T2D in KEGG. To ensure that our observations regarding the role of Cc on EGRN in teasing out biologically relevant pathway information cannot be reproduced from randomized data, we repeated the Cccalculations on degree-preserved randomized networks. We found that the Cc values of the significant pathways are higher on average for EGRN than randomized networks. The average of the means of the Cc distributions for randomized networks was 72.49, whereas the mean of the Cc distribution for the EGRN network was 121.32. The Mann–Whitney *U* *p*-values were between 9.54e-24 and 6.78e-11 (Fig. 2d). This both proves the utility of using the EGRN in conjunction with Cc and establishes Cc as an effective metric for prioritizing pathways.

**Fig. 3:**
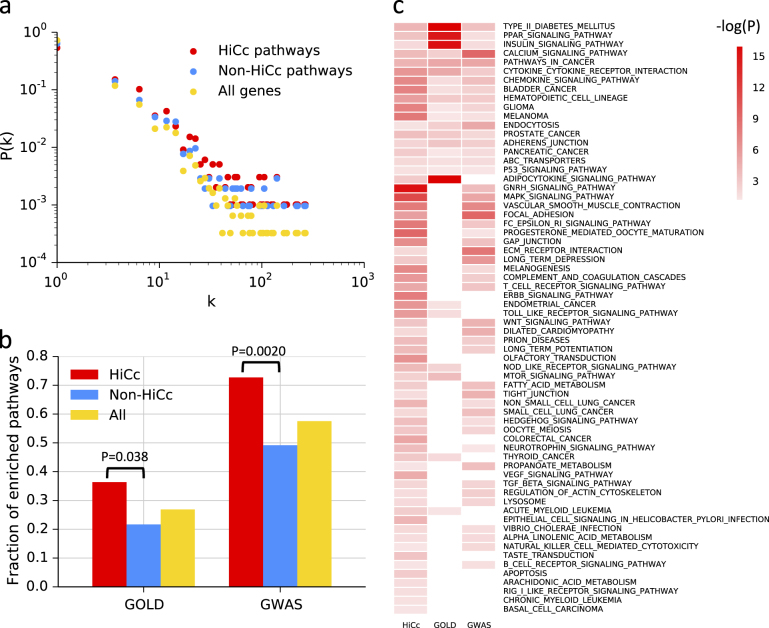
Properties and T2D relevance of high control centrality (HiCc) pathways. **a** Degree distributions P(k) of HiCc pathway genes, non-HiCc pathway genes, and all other genes in the EGRN. **b** The fraction of enriched pathways in the T2D GOLD and GWAS datasets, for HiCc pathways, non-HiCc pathways, and all pathways. **c** The 66 HiCc pathways and their enrichment in T2D-specific data sources

We next asked whether the genes within the high Cc pathways were also hubs, i.e., highly connected genes. We, therefore, compared the degree distributions of: (1) all genes in the EGRN, (2) all genes that are in any of the 66 significant HiCc pathways, and (3) all genes that are in any of the remaining 120 non-significant Cc pathways. Overall, we did not observe any significant differences between the three types of degree distributions as shown in Fig. 3a. Thus, both the significant and the non-significant pathways based on Cc values contain genes that have similar degree distribution in the EGRN, indicating an absence of bias towards hubs as high Cc genes.

To test the reliability and performance of the Cc approach as a means to glean key drivers of T2D, we compared it to other methods that identify the dysregulated subnetworks associated with a specific phenotype. We found that Cc is comparable to or higher performing than a number of established methods to find the dysregulated pathways, such as HotNet2^8^ in capturing “T2D-related” pathways, which are pathways significantly enriched in literature-mined T2D disease genes with experimental evidence from the DISEASES database,^16^ both on the EGRN and on generic networks (Supplementary Figure 3, see Supplementary Information for details on comparisons with other methods).

To further assess the performance of Cc as a network centrality measure, we carried out the T2D diabetes “T2D-related” pathway assessment on other centrality measures applied on the EGRN. We found that Cc is superior to all of the tested centrality measures in terms of the significance of overlap with T2D-related pathways, i.e., the HiCc pathways have a higher enrichment of T2D-related pathways than pathways with high centrality according to other centrality measures, including degree, closeness, eigenvector, betweenness, and PageRank centrality (Supplementary Table 1). The fact that Cc outperforms degree centrality also confirms our observation that Cc, which is not biased towards highly connected nodes, uncovers disease-related information that is independent of the “hubness” of a node.

### T2D relevance of the HiCc pathways in the EGRN

We hypothesized that if HiCc pathways contribute to the control of disease-related processes in T2D, they should be significantly enriched within T2D-specific -omics data. To test this hypothesis, we separated the HiCc pathways, i.e., pathways whose genes have significantly higher Cc values than the rest of the genes in EGRN, from those that are not HiCc. For both groups of pathways, as well as for the reference set of all KEGG pathways, we calculated how many of them are significantly enriched within (i) T2D (GOLD) genes from the type 2 diabetes genetic association database (T2DGADB)^17^ and (ii) a more recent and extended T2D GWAS dataset from a genome-wide meta-analysis (see 'Methods'). We observed a significant enrichment of HiCc pathways in the two datasets. In particular, the fraction of enriched pathways in GOLD data (50 pathways overall) was significantly higher for HiCc pathways (24 out of 66 pathways) than for non-HiCc pathways (26 out of 120 pathways), with a two-tailed Fisher’s exact *p*-value of 0.038. Similarly, the fraction of enriched pathways in the GWAS dataset (107 pathways overall) was significantly higher for HiCc (48 out of 66 HiCc pathways) than for non-HiCc pathways (59 out of 120 non-HiCc pathways), with a two-tailed Fisher’s exact *p*-value of 0.0020 (Fig. 3b). Overall, 55 HiCc pathways were enriched in T2D relevant omics data (Fig. 3c). Among the pathways that were the most significantly enriched in the -omics data were several whose relevance to T2D is well established, such as Type II diabetes mellitus, PPAR signaling, insulin signaling, calcium signaling and chemokine signaling pathways (Supplementary Table 2). The role of chemokine signaling is known in T2D, as islet inflammation is involved in the regulation of β-cell function and survival in T2D.^18^ This indicates that our approach captures pathways that have T2D relevance in an unbiased way. We corroborated the disease relevance of HiCc pathways on two other complex diseases: asthma and chronic obstructive pulmonary disease (COPD) (Supplementary Figures 4 and 5, see Supplementary Information for details).

### Validation of HiCc pathway genes

**Fig. 4:**
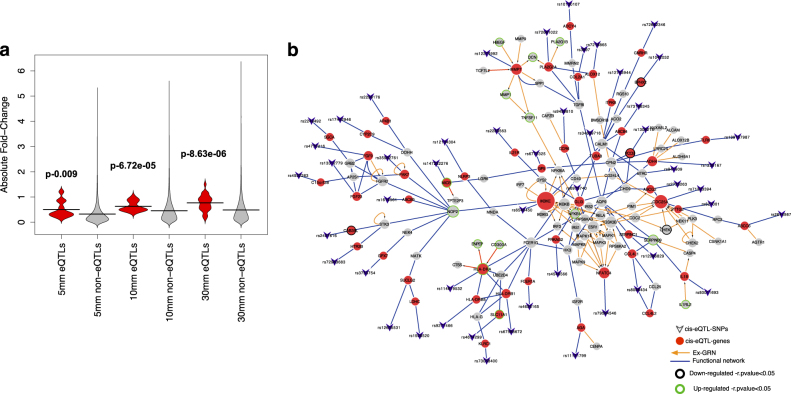
eQTLs and the functional network. **a** eQTL genes and glucose levels: we tested the fold change difference of eQTL genes vs non-eQTL genes in the transcriptomic data of rat islets pre-cultured at 2, 5, 10, and 30 mM glucose. eQTL genes are significantly changed in expression compared to non-eQTL genes. **b** Integrating EGRN and gene interaction networks with the eQTL-gene relationship associated with T2D. Most of the genes in the integrated module are up-regulated in T2D subjects (nodes in green)

Among the genes in the significant HiCc pathways, we found 51 eQTLs that pass the FDR < 1% threshold and 10 k permutation, as shown in Table 1, using an extended follow-up study to our original islet data, which consists of 89 pancreatic islet donors.^19^ In total, we observed a SNP within 250 kb up or downstream of the genes in 33 pathways. The enrichment of 33 pathways with the background distribution was significant (p- 6.618e-13, odds ratio: 7.49), which indicates that we were able to capture the genomic signals among the HiCc pathways. We next tested the fold-change difference of eQTL genes vs non-eQTL genes in the transcriptome data of rat islets pre-cultured with 2, 5, 10, and 30 mM glucose levels (GSE12817).^20^ We found that the eQTL genes have significantly higher fold change compared to non-eQTL genes in 5 mM (Mann-Whitney test *p* = 0.009), 10 mM (*p* = 6.72e-05), and 30 mM (*p* = 8.63e-06) glucose levels (Fig. 4a). This indicates the potential role of eQTL genes in β-cell function as these cells are regulated both acutely and chronically by the extracellular glucose concentration. By applying a greedy algorithm (Steiner tree) in an integrated network of EGRN and protein interaction network, we observed a single connected component of eQTL genes with few linkers (grey nodes) (Fig. 4b). This signifies that in reality a network environment is better characterized by the *local impact hypothesis*,^1^ indicating that perturbations are localized to the immediate vicinity of the perturbed genes that carry the eQTLs.

**Table 1:** cis-eQTL in HiCc pathway genes

SNP	Gene	*t*-stat	*p*-value	FDR	Permutation *p*-value	Quartile of expression (eQTL gene)	Closest = eQTL gene?	Closest gene	Nominal array eQTL	Genes on the array	eQTL in other tissues?	Pathways	Ex-GRN
rs2644987	ABCC6	4.543	1.82E-05	8.41E-03	1.00E-04	2	TRUE	ABCC6	YES	YES	yes	Abc_transporters	✔
rs2725263	ABCG2	−4.518	2.00E-05	9.09E-03	1.00E-04	2	TRUE	ABCG2	YES	YES		Abc_transporters	✔
rs73198345	ABCB4	5.214	1.28E-06	9.70E-04	1.00E-04	2	FALSE	ABCB1	YES	YES		Abc_transporters	
rs1619561	ABCB9	−4.572	1.63E-05	7.74E-03	1.00E-04	2	FALSE	MPHOSPH9	YES	YES	yes	Abc_transporters/lysosome	
rs4546566	PRKAG2	−4.851	5.50E-06	3.25E-03	1.00E-04	4	TRUE	PRKAG2		YES		Adipocytokine_signaling_pathway/insulin_signaling_pathway	
rs7216865	ALOX12	4.523	1.97E-05	8.96E-03	1.00E-04	2	TRUE	ALOX12		YES	yes	Arachidonic_acid_metabolism	✔
rs3753754	GPX7	−9.670	2.38E-15	2.30E-11	1.00E-04	3	TRUE	GPX7	YES	YES	yes	Arachidonic_acid_metabolism	
rs17442946	CYP2C9	4.991	3.15E-06	2.07E-03	1.00E-04	2	FALSE	CYP2C19		YES		Arachidonic_acid_metabolism	
rs1042032	EPHX2	4.611	1.40E-05	6.91E-03	1.00E-04	4	TRUE	EPHX2	YES	YES	yes	Arachidonic_acid_metabolism	
rs10130107	ADCY4	6.076	3.36E-08	4.09E-05	1.00E-04	2	FALSE	CMA1		YES		Calcium_signaling_pathway	
rs72983683	HTR2B	5.581	2.79E-07	2.54E-04	1.00E-04	2	FALSE	ARMC9	YES	YES		Calcium_signaling_pathway	
rs12743944	ITPKB	4.858	5.35E-06	3.17E-03	1.00E-04	2	FALSE	PARP1		YES		Calcium_signaling_pathway	
rs8066434	CCL4L1 + CCL4L2	5.236	1.17E-06	8.96E-04	1.00E-04	1	FALSE	CCL4				Chemokine_signaling_pathway	
rs9459810	CCR6	5.266	1.03E-06	8.07E-04	1.00E-04	1	FALSE	RNASET2		YES		Chemokine_signaling_pathway/cytokine_cytokine_receptor_interaction	✔
rs12086829	SERPINC1	5.326	8.08E-07	6.49E-04	1.00E-04	2	FALSE	RC3H1		YES	yes	Complement_and_coagulation_cascades	
rs80011693	IL18	−4.925	4.10E-06	2.54E-03	1.00E-04	3	TRUE	IL18	YES	YES	yes	Cytokine_cytokine_receptor_interaction	✔
rs2283563	IL21R	−5.139	1.74E-06	1.26E-03	1.00E-04	1	FALSE	IL4R		YES		Cytokine_cytokine_receptor_interaction	
rs2249492	SGCA	−4.802	6.68E-06	3.85E-03	1.00E-04	2	FALSE	MIR4315-2+ PLEKHM1P + MIR4315-1 + PLEKHM1		YES	yes	Dilated_cardiomyopathy	
rs35982761	PAK7	4.661	1.16E-05	5.99E-03	1.00E-04	2	TRUE	PAK7		YES		Erbb_signaling_pathway/focal_adhesion	✔
rs1230167	ADH4	−4.650	1.21E-05	6.19E-03	1.00E-04	1	FALSE	METAP1		YES		Fatty_acid_metabolism	✔
rs4656165	FCER1A	5.105	1.99E-06	1.41E-03	1.00E-04	1	FALSE	CADM3		YES		Fc_epsilon_ri_signaling_pathway	
rs3997	COL2A1	−5.418	5.52E-07	4.66E-04	1.00E-04	1	FALSE	SENP1		YES		Focal_adhesion	
rs34639716	TUBA8	4.802	6.68E-06	3.85E-03	1.00E-04	2	TRUE	TUBA8	YES	YES		Gap_junction	
rs72642346	GNRHR	4.932	3.98E-06	2.49E-03	1.00E-04	1	FALSE	UBA6		YES	yes	Gnrh_signaling_pathway	
rs6975740	GLI3	4.639	1.26E-05	6.38E-03	1.00E-04	2	FALSE	LOC285954	YES	YES		Hedgehog_signaling_pathway	
rs941909	BTRC	−4.506	2.09E-05	9.41E-03	1.00E-04	3	FALSE	LBX1		YES		Hedgehog_signaling_pathway	
rs114619532	HLA-DRA	−8.964	6.38E-14	4.76E-10	1.00E-04	2	FALSE	BTNL2		YES		Hematopoietic_cell_lineage	✔
rs9271466	HLA-DRB5	−12.218	2.07E-20	8.05E-16	1.00E-04	3	FALSE	HLA-DQA1	YES	YES	yes	Hematopoietic_cell_lineage	
rs67588672	HLA-DRB1	−9.685	2.22E-15	2.15E-11	1.00E-04	3	FALSE	HLA-DRB5			yes	Hematopoietic_cell_lineage	
rs6772325	GP5	4.656	1.18E-05	6.08E-03	1.00E-04	1	FALSE	ATP13A3		YES		Hematopoietic_cell_lineage	
rs72661022	PLA2G2A	4.921	4.16E-06	2.57E-03	1.00E-04	3	TRUE	PLA2G2A	YES	YES		Long_term_potentiation	✔
rs11131799	AGA	5.432	5.22E-07	4.43E-04	1.00E-04	4	TRUE	AGA	YES	YES	yes	Lysosome	✔
rs4932263	AP3S2 + C15orf38 + C15orf38-AP3S2	−9.449	6.65E-15	5.93E-11	1.00E-04	4	TRUE	AP3S2	YES	YES	yes	Lysosome	
rs2296176	AP4B1	−4.799	6.75E-06	3.88E-03	1.00E-04	3	FALSE	BCL2L15	YES	YES	yes	Lysosome	
rs4674299	SLC11A1	4.694	1.02E-05	5.39E-03	1.00E-04	2	FALSE	C2orf62		YES		Lysosome	
rs13312779	FGF23	6.902	8.66E-10	1.45E-06	1.00E-04	1	TRUE	FGF23		YES		Mapk_signaling_pathway	
rs4770216	FGF9	4.830	5.98E-06	3.50E-03	1.00E-04	2	TRUE	FGF9	YES	YES		Mapk_signaling_pathway/regulation_of_actin_cytoskeleton	✔
rs2407616	CAB39L	−4.858	5.36E-06	3.18E-03	1.00E-04	3	TRUE	CAB39L	YES	YES		Mtor_signaling_pathway	
rs147232276	MICB	−6.252	1.56E-08	2.05E-05	1.00E-04	1	TRUE	MICB		YES	yes	Natural_killer_cell_mediated_cytotoxicity	✔
rs73058400	KLRC1	6.995	5.68E-10	9.97E-07	1.00E-04	1	TRUE	KLRC1		YES		Natural_killer_cell_mediated_cytotoxicity	
rs12134304	NLRP3	5.453	4.78E-07	4.10E-04	1.00E-04	2	TRUE	NLRP3	YES	YES		Nod_like_receptor_signaling_pathway	
rs627001	PTTG2	4.548	1.78E-05	8.32E-03	1.00E-04	1	TRUE	PTTG2		YES		Oocyte_meiosis	✔
rs71323394	CDC25A	4.937	3.92E-06	2.46E-03	1.00E-04	2	FALSE	SPINK8	YES	YES	yes	Progesterone_mediated_oocyte_maturation	✔
rs1506520	LDHC	−25.886	4.44E-42	3.86E-36	1.00E-04	2	TRUE	LDHC	YES	YES	yes	Propanoate_metabolism	
rs12635531	SUCLG2	4.787	7.06E-06	4.03E-03	1.00E-04	4	TRUE	SUCLG2	YES	YES	yes	Propanoate_metabolism	
rs13082184	PCCB	−4.771	7.52E-06	4.21E-03	1.00E-04	4	FALSE	STAG1	YES	YES		Propanoate_metabolism	
rs6540450	IKBKE	−4.966	3.49E-06	2.24E-03	1.00E-04	2	FALSE	RASSF5	YES	YES		RIG_i_like_receptor_signaling_pathway	✔
rs139157987	TLR6	6.553	4.14E-09	6.14E-06	1.00E-04	2	TRUE	TLR6	YES	YES		Toll_like_receptor_signaling_pathway	
rs12274992	MMP7	4.914	4.29E-06	2.63E-03	1.00E-04	4	FALSE	MMP27	YES	YES		Wnt_signaling_pathway	✔
rs79584546	NFATC4	5.677	1.86E-07	1.78E-04	1.00E-04	2	FALSE	CMA1		YES		Wnt_signaling_pathway/B_cell_receptor_signaling_pathway/MAPK_signaling_pathway/T_cell_receptor_signaling_pathway	✔

### New mechanistic connections in T2D

**Fig. 5:**
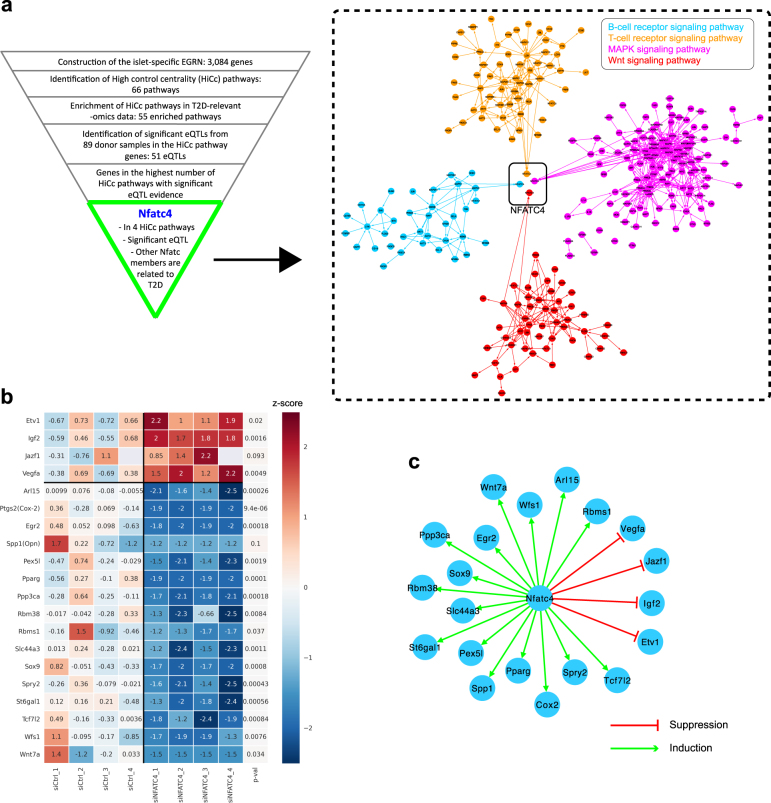
Nfatc4 *in vitro* validation. **a** Nfatc4 is at the intersection of four HiCc pathways, namely B-cell receptor signaling pathway, T-cell receptor signaling pathway, MAPK signaling pathway, and Wnt signaling pathway. **b** The effect of silencing of Nfatc4 on putative downstream genes. Colors indicate the *z*-score, which was calculated across all samples per gene and is shown relative to the average *z*-score of the control samples. *p*-values were obtained by a two-sided *t*-test for two independent samples. **c** The network of the putative downstream effect of Nfatc4 validated by in vitro silencing experiments

To find the connection between the HiCc pathways and T2D-associated genes, we focused on an eQTL SNP (rs79584546) associated with the expression of the gene NFATC4 (*q*-value = 1.78E-04), which is associated with four HiCc pathways: Wnt signaling, B-cell receptor signaling, MAPK signaling, and T-cell receptor signaling pathways (Fig. 5a). We asked whether the T2D-related genes implicated in GWAS and literature are downstream targets of NFATC4. This might help in explaining their role in four HiCc pathways. The NFATC4 gene interacts with PPARG, and different MAP kinases (MAPK1, MAPK3, MAPK8, MAPK9, MAPK14) (Supplementary Figure 6). It is known that ablation of NFATC4 increases insulin sensitivity, in part, by sustained activation of the insulin-signaling pathway.^21^ We, therefore, next explored the downstream effect of transcriptional targets of NFATC4.

NFATC4 has been reported to be a possible target for up-regulated transcription by TGF-alpha.^22^ Therefore, we used TGF-alpha to augment or induce Nfatc4 mRNA transcription during a glucose challenge in functional rat pancreatic beta islet cells in vitro. Nfatc4 was effectively silenced (see 'Methods' for details on the in vitro silencing experiments) as seen in real time qPCR with four different rat-specific Nfatc4 probes (Supplementary Figure 7a-d). In order to assess the downstream effect of Nfatc4, we gathered putative T2D candidate genes regulated by the NFAT family members from GWAS and literature.^23^ In particular, we selected 13 highly up-regulated and down-regulated transcriptional targets of Nfatc1 and Nfatc2, namely Etv1, Jazf1, Pparg, Vegfa, Arl15, Pex5l, Rbm38, Rbms1, Slc44a3, Spry2, St6gal1, Tcf7l2, and Wfs1, based on a recent report^23^ that they inhibit the expression of the first four genes while promoting the expression of the latter nine genes. We supplemented this list with seven putative transcriptional targets of Nfatc4, namely Cox2 (Ptgs2), Egr2, Igf2, Opn (Spp1), Ppp3ca, Sox9 and Wnt7a, through literature search via the MetaCore platform [https://portal.genego.com/]. The silencing of Nfatc4 in rat islet cell lines resulted in increased expression of, Etv1, Igf2, Jazf1, and Vegfa mRNA, compared to control siRNA treatment. In contrast, Arl15, Cox2, Egr2, Opn, Pexl5, Pparg, Ppp3ca, Rbm38, Rbms1, Slc44a3, Sox9, Spry2, St6gal1, Tcf7l2, Wfs1, and Wnt7a, all resulted in decreased mRNA expression post Nfatc4 silencing (Fig. 5b).

The silencing of Nfatc4 in rat islet cell lines, thus displays reduced expression of 16 downstream T2D candidate genes and increased expression of four downstream T2D candidate genes. To complete the circle, we went back to the human islet data and assessed correlation of Nfatc4 expression with the aforementioned genes. We found that NFATC4 expression was positively correlated with ETV1, VEGFA, EGR2, RBM38, SOX9, ST6GAL1, TCF7L2, WFS1, and WNT7A and negatively correlated with SPRY2 (Supplementary Figure 8 and Supplementary Table 6). This indicated the potential influence of NFATC4 expression on the expression of the above genes in human pancreatic islets as well. NFATC4 expression also positively correlated with HbA1C levels, indicative of some effect on glycemic status. The mechanistic connection between NFATC4 and TCF7L2 is particularly of interest as TCF7L2 has been established as a major T2D susceptibility gene.^23,24^ TCF7L2 is also a member of two of our HiCc pathways, namely B-cell receptor signaling and Wnt signaling pathways. This indicates the possibility of finding further unexplored connections between the members of HiCc pathways within the context of specific diseases. Overall, the approach helps not only in identifying the potential dysregulated pathways, but also establishes the downstream regulation by NFATC4 in four important T2D pathways (Fig. 5).

## Discussion

By exploiting the topological measures of cellular re-wiring associated with disease progression, it is possible to identify new disease genes and pathways.^1,2,6^ With the advances in control theory, and control principles becoming an important consideration in many disciplines, including disease biology and biological network analysis,^25–27^ network dynamics and regulation also provide opportunities to identify key regulatory genes in health and disease. Here, we exploited the Cc measure to identify key pathways that could drive the islet regulatory network in T2D. We established a framework to find the pathways that might be related to the underlying hierarchical structure of disease regulation and might add a new dimension in finding dysregulated pathways compared to a number of established methods like HotNet2.^8^ We identified 66 pathways as statistically significant in the analysis and were able to identify known T2D pathways (*p* = 5.76E-05) among the top gene list in our analysis, which validates the approach as a means to capture disease relevant pathways. The pathways captured in our analysis were also enriched in T2D relevant omics sources. Furthermore, eQTL analysis helped in pruning the HiCc pathways genes by identifying the variants actually affecting the gene expression levels of these genes (i.e., cis-eQTLs). These eQTL genes showed glucose-induced changes in the islets.

Moreover, the application of our approach to other complex diseases (asthma and COPD) suggests that it can be generalized, and could be used in the context of other diseases in the future to identify the key driver pathways.

Finally, we experimentally validated the process by which a particular eQTL gene (NFATC4) regulates the expression of numerous putative downstream T2D candidate genes of two other genes of the same family, NFATC1 and NFATC2, which were also shown to regulate T2D related genes by previous studies.^23^ In particular, Nfatc4 silencing results confirmed similar transcription regulation pattern for these genes except for Igf2, which showed an opposite effect relative to the siControl condition. Results also demonstrated that Nfatc4 increases gene expression of Pparg, Tcf7l2, and Wfs1 which are genes already reported to be associated with T2D as well as activation of the Wnt pathway as predicted by systems genetics approach that is subsequently validated in vitro.^28^ However, silencing Nfatc4 also appears to have a tendency to inhibit osteopontin (Opn). We have previously demonstrated that glucose dependent insulinotropic polypeptide (GIP) stimulates expression of OPN in human islets where OPN exerts protection against cytokine-induced apoptosis.^29^ The connection between NFATC4 and TCF7L2, which has not been reported previously in the literature, is particularly important as it adds to the mechanistic information on two pathways (Wnt signaling and B-cell signaling pathways) that were found to have HiCc in the T2D pathobiologic context. Owing to this new connection, NFATC4 and TCF7L2 also emerge as potential players in the pathway communication between the T-cell receptor signaling, MAPK signaling, Wnt signaling, and B-cell signaling pathways. Overall, the positive experimental validation of our model shows the utility of the Cc approach in pathway prioritization. In particular, it may pave the way for discovering hitherto uncovered cross-talk between pathways.

These results might help us understand better the controllability of complex networks and provide a basis for designing an efficient strategy for optimizing (normal) network control. There are, however, outstanding annotation and methodological challenges remaining, including low-resolution pathway-based knowledge, limited cell type-specific information, and incomplete annotation of next-generation pathways. Despite these hurdles, as the number and type of functional annotations increase, coupled with technological advances in analytical methods that provide better guidance for the utility of pathway analysis, confidence in the results will likely improve. Although the approach has been demonstrated using pancreatic islet gene-expression data, it can be used to interpret pathways for other complex diseases. Overall, controllability-based network analysis may be of broad use in dissecting complex diseases and in discovering novel therapeutic targets in this coming era of systems medicine.

## Methods

### Construction of GRN

We constructed a disease GRN by integrating gene expression data from human pancreatic islets together with known information about transcription factor binding sites. We used gene expression data from islets from 63 cadaver donors provided by the Nordic Islet Transplantation Programme (http://www.nordicislets.org) (Supplementary Table 3). Islets were obtained from 54 nondiabetic donors (25 females, 29 males, age 59 ± 9, BMI 25.9 ± 3.5, HbA1c 5.5 ± 1.1) and nine T2D donors (four females, five males, age 57 ± 4, BMI 28.5 ± 4.5, HbA1c 7.2 ± 1.1). All procedures were approved by the ethics committee at Lund University. Purity of islets was assessed by dithizone staining, while measurement of DNA content and estimation of the contribution of exocrine and endocrine tissue were assessed as previously described.^3^ Gene expression was assayed using Affymetrix Human Gene 1.0 ST arrays. We normalized the data by robust multiarray averaging (RMA) and a custom Chip Description File (CDF) from the Michigan Microarray Lab (http://brainarray.mbni.med.umich.edu, Version 13) which helps estimate gene-level expression more accurately by summarizing probe sequences using up-to-date gene annotations.

To construct the GRN, we first performed feature selection on the genes using a three-step procedure. First, we computed the variance of the expression of each gene on the array across all 63 samples, and selected the top 2000 most variable genes to add tissue-specific genes that are expressed and active in human islets. Next, we added the nearest genes of the 48 risk SNPs that have been associated with T2D through GWAS (Supplementary Table 4) and that were also represented on the array. Finally, we supplemented these genes with differentially expressed genes. To do this, we used the LIMMA package in R^30^ to compute the B-statistic for differential expression between the following phenotypic groups: (1) diabetic vs non-diabetic, and (2) low vs high levels of HbA1c. These comparisons were carried out between 63 patients with and without T2D for (1) and donors with HbA1c < 6% and >6% from the human islets mRNA data set for (2). Because very few genes passed statistical significance after Benjamini-Hochberg adjustment for multiple testing, we used a liberal *p* < 0.05 threshold for the nominal (unadjusted) *p*-value to obtain a permissive list of potential disease signature genes, as has been done previously.^2^ These genes were ranked according to the average of the two B-statistics, and the top 464 genes that were not already in the GWAS or high-variance gene sets were chosen to supplement the gene list up to a total of 2500 genes.

The final list of 2500 genes, together with the corresponding gene expression values over all 63 patients, was used as input to build the GRN. To infer the network, we used the R/Bioconductor package *predictionet* to combine a set of known regulatory interactions together with gene expression data via mutual information and causality inference.^31^ Briefly, *predictionet* first uses the maximum relevance minimum redundancy (MRMR) approach to select the optimal non-redundant set of parents (or likely regulators) for each gene. In the first iteration of MRMR, each target gene is assigned a parent node by maximizing the mutual information between the expression profiles of the target gene and the parent gene. In each successive iteration, another parent is added by maximizing the mutual information between the new parent and the target gene while minimizing the mutual information of the new parent with the existing parents. This process is repeated until the maximum number of possible parents is reached for each target gene; we allowed a maximum of three parents, which is the default value. Next, each parent node is assigned a causality score, which ranges from −1 to 1, based on whether it exhibits conditional mutual information with the other parent nodes, anchoring it as part of a causal “v-structure.” In the last step, one computes a weighted sum of the network of known regulatory interactions with the causality-based network, with the weight parameter chosen by the user. All edges with a final score greater than zero are retained to populate the inferred network. In our case, as prior evidence we used known regulatory interactions from the TRANSFAC database and applied a weighting parameter of 0.75 to combine the MRMR and prior-based edges.

### Extended gene-regulatory network (EGRN)

A gene can be involved in various interactions, and its role, and consequently its centrality, can vary across different biological networks. In order to obtain a higher resolution understanding of the signaling relationship in the GRN, we added kinase (http://www.phosphosite.org) and signaling events to the model (Signalink database).^32^ This addition was done in order to add potential downstream signaling events by the nodes in the GRN. We call the full network, including transcriptional edges and signaling edges, the EGRN.

### Ranking the KEGG pathways using the Cc measure—HiCc pathways

We started with an un-weighted directed EGRN *G* = (*V*, *E*) with *N* = |*V*| nodes and *L* = |*E*| links. The Cc of node *i*, denoted as *C*_c_(*i*), is defined to be the generic dimension of controllable subspace or the size of controllable subsystems if we control node *i* only.^15^ Hence, *C*_c_(*i*) captures the “power” of node *i* in controlling the whole network. For example, a simple network of *N* = 7 nodes is shown in Fig.1b. When we control node *x*_1_ only, the controlled network is represented by a directed network with an input node *u*_1_ connected to *x*_1_. The dimension of the controllable subspace by controlling nodes *x*_1_ is six, corresponding to the largest number of edges in all stem-cycle disjoint subgraphs (an example is shown in red in Fig. 1), where “stem” is defined as a directed path starting from an input node, so that no nodes appear more than once in it. Hence, the Cc of node *x*_1_ is C_c_(1) = 6. In general, the Cc of any node in a directed network can be calculated by solving a linear programming problem.^15,33,34^ The assumption was that if a pathway or module includes genes with high C_c_ values, it might be higher in the hierarchy and must regulate other downstream pathways with on average, lower C_c_ values. To test this hypothesis, for each pathway in the KEGG database, we identify the pathways with significantly greater C_c_ values compared to others. To calculate the statistical significance based on the C_c_ values of the genes representing the particular pathway, we use the Mann–Whitney *U* test with a cutoff *p*-value < 0.05. A typical problem with pathway analysis methods is bias toward the enrichment of cancer-related pathways as these pathways have been studied more intensively. We, therefore, focus only on the non-cancer HiCc pathways.

### T2D relevance of the HiCc pathways in the EGRN

We next evaluated the relative enrichment of HiCc pathways genes across two T2D-specific datasets:

i. *Disease gene set*: T2D genetic association database (T2DGADB) aims to provide specialized information on the genetic risk factors involved in the development of T2D. Seven hundred one publications in the T2D case-control genetic studies for the development of the disease were extracted,^35^ which was defined as the gold standard gene set. Overall, this dataset contained 143 genes.
ii. *Genomics:* This genome-wide meta-analysis (“DIAGRAMv3”) includes data from 12171 cases and 56,862 controls of European descent imputed up to 2.5 million autosomal SNPs.^36^ We computed a single *p*-value for each gene in the interactome by the VEGAS method using the whole GWAS data set.^37^ We considered the genes with uncorrected *p*-value < 0.01 in our analysis, resulting in 1308 genes. There was little overlap between (i) and (ii) gene sets. Moreover, to avoid circularity, we exclude the 48 genes implicated in GWAS that were used in the construction of EGRN from this dataset.

The enrichment of HiCc, non-HiCc and all pathway genes with the above gene sets was calculated through Fisher’s Exact test.

### eQTL analysis of HiCc pathway genes

One of the major findings from the T2D GWAS is that most of the trait-associated SNPs are located in intronic, intergenic, or other non-coding regions of the genome.^38^ As many SNPs are located in noncoding regions, suggesting they may influence gene expression, we analyzed whether any SNP within 250 kb of HiCc pathway genes (cis) would influence their gene expression (eQTL). We used a linear model adjusting for age and sex as implemented in the R Matrix eQTL package. Genotyping was performed on the Illumina HumanOmniExpress 12v1C chips, and all the samples passed standard genotype QC metrics. Genotypes were imputed to 1000 Genomes data, using IMPUTE2 and SHAPEIT.

The transcriptomic data from rat pancreatic islet after culture in low, intermediate and high glucose was retrieved from Gene Expression Omnibus (GEO-GSE12817). We performed differential expression analysis between 2 and 10, 2 and 30, 5 and 10, 5 and 30 or 10 and 30 mmol/l glucose. We used the limma R package (ver 3.10.1) for the differential expression analysis. We compared the fold change (absolute log) of eQTL genes to all other differentially expressed genes and computed the *p*-values by applying Mann–Whitney *U* test.

### Functional network and eQTL-HiCc pathway genes

To evaluate the impact of genes in the vicinity of eQTL in different sources of network data, we used HumanNet gene-interaction data. HumanNet uses a Näive Bayesian approach to weight different types of evidence together into a single interaction score focusing on data collected in humans, yeast, worms, and flies.^39^ The hypothesis we tested was that if the gene products (e.g., proteins) linked to the same disease phenotype interact with each other more often than randomly linked gene products^40–42^ and cluster in the same network neighborhood, then eQTL genes must be connected through a single component in the gene interaction network. To find the minimum number of genes that can connect the eQTL into a connected component in the gene-interaction network, we applied the Steiner tree algorithm using the Klein-Ravi approximation^43^ implemented in the GenRev Python package (https://bioinfo.uth.edu/GenRev.html),^44^ which is a node-weighted variant of the Steiner tree problem and uses a greedy search strategy to iteratively merge the terminal (or seed) genes into one large tree by first mapping terminals to the network to see if they have any direct interactions.

### **Nfatc4 silencing and stimulation experimentsin vitro**

To validate Nfatc4 effect on putative downstream T2D candidate genes, Nfatc4 mRNA expression was silenced in clonally derived rat pancreatic β-islet cell line INS-1 (832/13), a generous contribution from Dr. Rohit Kulkarni. Cells were maintained on RPMI 1640, 10% fetal calf serum, 10 mM HEPES, 2 mM L-glutamine, 1 mM sodium pyruvate, and 0.05 mM 2-mercaptoethanol (Thermo Fisher) supplemented with penicillin (100 Units/ml) and streptomycin (100 μg/ml) (Pen/Strep). We transfected rat specific Nfatc4 siRNA (Rat Nfatc4 ON-TARGET Smart Pool siRNA, GE Dharmacon) using Lipofectamine RNAiMAX (Thermo Fisher) as per manufacturer’s recommendation. For controls, we employed ON-TARGETplus Non-Targeting Control Pool (GE Dharmacon) using the same concentration as the test siRNA. Silencing was carried out in each well containing 60% confluent cells in culture and were exposed to transfecting medium for 48 h at 37 °C in a humidified atmosphere containing 95% air and 5% CO_2_ in the presence of 20 pmol siRNA per well in 12-well cell culture microplates.

After silencing, cells were allowed to recover for 16 h in regular growth media followed by stimulation of Nfatc4 signaling by TGF alpha (TGF-a) at 50 ng/ml as described in another publication^22^ for 6 h. Subsequently, cells were washed with HBSS once, then exposed to 17.3 mM glucose in HBSS for 1.5 h. RNA from cells were harvested using column isolation (GE Healthcare Life Sciences). Putative genes downstream of Nfatc4 were tested using probes from Taqman Gene Expression Assay system (Thermo Fisher) listed in Supplementary Table 5. Real time PCR was done with Biomark HD (Fluidigm) thermocycler. Quadruplicates were used per condition.

### Expression correlation of NFATC4 with putative downstream genes in human islet cells

RNA sequencing was performed on the Hi-seq as described previously.^19^ Alignments were performed using STAR and gene counts were assessed using featureCounts. Spearman correlation was used to assess the relationships between NFATC4 expression and ETV1, IGF2, JAZF1, VEGFA, PTGS2/COX2, EGR2, SPP1 (OPN), PPARG, PPP3CA, RBM38, RBMS1, SOX9, SPRY2, ST6GAL1, TCF7L2, WFS1, and WNT7A.

### Data availability

The data that support the findings of this study are available from the corresponding author upon reasonable request.

## Electronic supplementary material

Supplementary information

## Electronic supplementary material

**Supplementary information** accompanies the paper on the *npj Systems Biology and Applications* website (10.1038/s41540-018-0057-0).
