# Supplementary material for: Controllability in an islet specific regulatory network identifies the transcriptional factor NFATC4, which regulates Type 2 Diabetes associated genes
Source: NPJ Syst Biol Appl. 2018 Jul 3;4:25. doi: 10.1038/s41540-018-0057-0 (PMC6028434; doi:10.1038/s41540-018-0057-0)
Supplement: Supplementary file 1 — Supplementary information [file 41540_2018_57_MOESM1_ESM.pdf]

Controllability in an islet specific regulatory network identifies the transcriptional factor NFATC4, which regulates Type 2 Diabetes associated genes

Supplementary Notes

Comparison of HiCc approach with other methodologies

As a complementary approach and a performance benchmark, we compared the output of the control centrality approach to other methods that identify the dysregulated subnetworks associated with a specific phenotype. We first extracted the literature-mined T2D disease genes with experimental evidence from the DISEASES database [1]. Next, we performed pathway enrichment analysis on this set of disease genes to be subsequently used as "T2D-related pathways." This resulted in 32 T2D-related pathways out of the 186 KEGG pathways. The overlap of the 66 HiCc pathways with the 32 T2D related pathways is 21 (p-value=0.00018 and -log P = 8.62, two-sided Fisher's Exact test; Jaccard index=0.27). We first compared these values to those of jActiveModules [2]. The p-value of HiCc is higher (z-score=0.94) than the mean of the p-value distribution (mean=0.25, std=0.03) of the jActive modules. Similarly, although the HiCc -log P value is not significantly different from the Jactive modules' –log P distribution (z-score = 0.94), it is higher than the mean of the distribution (mean= 6.53, std = 2.23) (Supplementary Figures 3a & b). Thus, overall, HiCc yields comparable results to jActive and, on average, HiCc gives better results than jActive (both Jaccard and -log P). We also looked at the number of T2D-related pathways captured by HiCc versus jActive, this time considering the union of all genes captured by the 66 jActive modules, amounting to 727 genes. These 727 genes resulted in 93 significantly enriched pathways. Out of these 93 significantly enriched jActive pathways, 26 are T2D related pathways, corresponding to –log P=8.79 (Fisher's Exact test). Once again, we observed that the two methods are comparable (-log P=8.79 and 8.62 for Jactive and HiCc, respectively).

We repeated the same procedure with HotNet2 [3]. We compared the gene set enrichment with respect to our T2D-related pathway set of genes. We found that 26 out of 1,012 genes related to the 66 HiCc pathways are captured as T2D-related pathways, giving a p-value for enrichment of 7.35e-6 by Fisher's Exact test. This p-value is lower than the enrichment for the largest HotNet2 clusters for all delta values (Supplementary Figure 3c), showing that the control centrality measure is able to capture more relevant disease-related genes and pathways compared to HotNet2 clusters.

In order to determine the network specificity of this analysis, i.e., the uniqueness of the extended regulatory network (EGRN) in producing biologically meaningful results when used in conjunction with the HiCc method, we compared with two additional methods which take generic networks as input, namely minimum dominating sets (MDSets) [4] and NetWAS [5]. These prioritization methods were applied on a predefined protein-protein network and a pancreatic islet specific protein interaction network (NetWAS). In both cases, HiCc performed better in terms of capturing T2D related pathways. In proteins belonging to the MDset, 111 pathways were enriched, 26 of which are T2D related. This resulted in p-value=0.0058 and –log P= 5.14. Similarly for NetWAS, out of its 60 significant pathways, 17 are T2D related (-log P=4.92). Comparing these values with 21/66 T2D related pathways of HiCc, applied on the EGRN, (-log P = 8.62), we found that applying HiCc that uses EGRN as the underlying network is significantly better at capturing T2D related pathways than applying methods that use generic networks.

HiCc pathways of Asthma and Chronic Obstructive Pulmonary Disease (COPD)

To extend the applicability of the control centrality approach to identifying key driver pathways in other complex diseases, we chose two complex chronic conditions, asthma and COPD. To create the asthma and COPD GRNs, we followed a very similar feature selection procedure as in the case of diabetes. We first selected the top 2000 genes with the highest variance across all samples. We then added in a manually curated list of genes known to be associated with each disease through

GWAS and other genetic and experimental studies: For the inference of the asthma GRN, we used gene expression data from airway epithelial cells of asthmatic and control subjects [6] along with GWAS-implicated genes from expert-curated lists and the GRASP database [7]. For the COPD GRN, we used gene expression data from severe COPD lung tissue from a previous study [8], along with COPD GWAS-implicated genes from two recent studies [9,10]. Finally, we used LIMMA to compare gene expression profiles of patients with asthma (or COPD) against matched healthy controls and adjusted the p-values using the Benjamini-Hochberg method. We then extracted the differentially expressed genes with $P_{\text{adj}} < 0.05$ and combined them with the GWAS-implicated genes and high-variance genes to produce the final list of input genes for network inference. The *predictionet* package was used to infer the final networks using the same exact priors and weighting scheme as described above in the diabetes analysis.

The resulting GRNs consisted of 846 and 774 genes for asthma and COPD, respectively. We then built the EGRNs by incorporating signaling [11] and kinase-substrate interactions [12]. Overall, the resulting EGRNs consisted of 2,004 and 1,335 edges for asthma and COPD, respectively. We then calculated the control centrality (Cc) values of every gene in each EGRN and determined the HiCc pathways by comparing their Cc distributions with the background Cc distribution using a two-sided Mann-Whitney U test, as detailed in the Methods section. This resulted in 32 HiCc pathways for asthma and 42 HiCc pathways for COPD (Mann-Whitney U test p-value < 0.05). To test the hypothesis that HiCc pathways offer an advantage in capturing disease-related signatures, we divided the 186 KEGG pathways in the Molecular Signatures Database (MSigDB) [13] into HiCc and non-HiCc pathways. The degree distributions of the genes within HiCc pathways and non-HiCc pathways showed no distinction, suggesting once again that control centrality is not biased towards highly connected nodes, or hubs, for asthma and COPD alike (Supplementary Figures 4a and 5a).

To validate the biological significance of the HiCc pathways, we looked for their

enrichment in asthma- and COPD-specific gene sets derived from literature and genome-wide association studies (GWAS). In particular, the literature-curated ("GOLD") disease genes were taken from the DisGeNet [14] database with a score cutoff of 0.15. The GWAS-implicated disease genes were obtained from meta-analyses of asthma [15] and COPD [16] GWA studies. To avoid circularity, the set of GWAS-implicated genes that were used in the construction of the asthma and COPD GRNs were excluded from these validation datasets.

For both asthma and COPD, we observed a significant enrichment of HiCc pathways in the two datasets compared to non-HiCc pathways. For asthma, the fraction of enriched pathways in GWAS data (53 pathways overall) was significantly higher for HiCc pathways (16 out of 32 HiCc pathways) than for non-HiCc pathways (37 out of non-HiCc 154 pathways), with a two-tailed Fisher's exact p-value of 0.0048, whereas the fraction of enriched pathways in GOLD data (55 pathways overall) was significantly higher for HiCc pathways (15 out of 32 HiCc pathways) than for non-HiCc pathways (40 out of non-HiCc 154 pathways), with a two-tailed Fisher's exact p-value of 0.0315. For COPD, the fraction of enriched pathways in GWAS data (45 pathways overall) was significantly higher for HiCc pathways (17 out of 42 HiCc pathways) than for non-HiCc pathways (28 out of non-HiCc 144 pathways), with a two-tailed Fisher's exact p-value of 0.0076, whereas the fraction of enriched pathways in GOLD data (47 pathways overall) was significantly higher for HiCc pathways (18 out of 42 HiCc pathways) than for non-HiCc pathways (29 out of non-HiCc 144 pathways), with a two-tailed Fisher's exact p-value of 0.0045 (Supplementary Figures 4b and 5b).

Inspecting the HiCc pathways from the asthma EGRN, we found that they recapitulate the GWAS and literature-based knowledge on asthma-related pathways. Pathways that have been linked to asthma previously, such as JAK-STAT [17], MAPK [18], TGF-β [19], ERBB [20], Wnt [21] and neurotrophin[22] signaling, adherens junction [23], apoptosis [24], and natural killer cell mediated cytotoxicity [25] pathways were captured as HiCc pathways alongside GWAS and literature-based genes.

Furthermore, the asthma pathways that were only captured by HiCc showed many interesting and recently explored connections such as long-term potentiation [26] and long-term depression [27,28], olfactory transduction [29], glioma [30], Notch [31] signaling, and regulation of actin cytoskeleton [32] (Supplementary Figures 4c and 5c).

The COPD HiCc pathways were also confirmed by literature meanwhile providing possibly novel insights. Pathways previously associated with COPD, such as MAPK [33], chemokine [34], T cell receptor [35], ECM receptor [36] and neurotrophin [37] signaling, cytokine-cytokine receptor interaction [38], Fc gamma receptor mediated phagocytosis [39], NK cell mediated cytotoxicity [40], focal adhesion [41], inositol phosphate metabolism [42], small [43] and non-small [44] cell lung cancer, apoptosis [45], cell cycle [46], leukocyte transendothelial migration [47], and autoimmune thyroid disease [33] pathways. COPD-related pathways that were captured solely by HiCc pathways included VEGF [48], mTOR [49,50], and ERBB [51], B-cell receptor [52], and p53 [53] signaling, as well as proteasome [54] and base excision repair [55] pathways.

Supplementary Methods

Benchmarking with other subnetwork identification methods

JActiveModules: We retrieved the connected subnetworks of the EGRN that show significant changes in expression using the JActiveModules plugin of Cytoscape. We set the number of subnetworks to 66 for fair comparison with the number of HiCc pathways and selected an overlap threshold of 0.8.

HotNet2: We extracted the significantly altered subnetworks using a heatfile of expression p-values (multiple testing adjusted), which served as the input for the heat diffusion kernel used by this method. Parameter (delta) selection was done automatically by the algorithm via permuted networks, outputting 4 subnetwork configurations corresponding to 4 delta values. As subnetwork configurations are dominated by one large subnetwork and numerous very small (size < 10)

subnetworks, we performed the gene set enrichment analyses on the largest subnetwork.

MDSets: The minimum dominating set (MDSet) of nodes is the smallest subset of nodes in a network from which every remaining non-dominating set node can be reached in one step. We determined the MDSet on the network of protein-protein interactions, which is a binary integer programming problem, using a linear programming-based branch-and-bound algorithm, as was done in [4].

NetWAS: We applied a z-score cutoff of 0.8, which resulted in 902 genes that yielded 60 significantly enriched pathways. DIAGRAMv3 GWAS data was used in the analysis.

References

1. Pletscher-Frankild, S., Pallejà, A., Tsafou, K., Binder, J. X. & Jensen, L. J. DISEASES: Text mining and data integration of disease–gene associations. *Methods* **74,** 83–89 (2015).

2. Ideker, T., Ozier, O., Schwikowski, B. & Siegel, A. F. Discovering regulatory and signalling circuits in molecular interaction networks. *Bioinformatics* **18 Suppl 1,** S233-40 (2002).

3. Leiserson, M. D. M. *et al.* Pan-cancer network analysis identifies combinations of rare somatic mutations across pathways and protein complexes. *Nat. Genet.* **47,** 106–114 (2015).

4. Kim, Y.-A., Wuchty, S. & Przytycka, T. M. Identifying Causal Genes and Dysregulated Pathways in Complex Diseases. *PLoS Comput. Biol.* **7,** e1001095 (2011).

5. Greene, C. S. *et al.* Understanding multicellular function and disease with

human tissue-specific networks. *Nat. Genet.* **47,** 569–576 (2015).

6. Woodruff, P. G. *et al.* Genome-wide profiling identifies epithelial cell genes associated with asthma and with treatment response to corticosteroids. *Proc. Natl. Acad. Sci.* **104,** 15858–15863 (2007).

7. Leslie, R., O'Donnell, C. J. & Johnson, A. D. GRASP: analysis of genotype-phenotype results from 1390 genome-wide association studies and corresponding open access database. *Bioinformatics* **30,** i185–i194 (2014).

8. Morrow, J. D. *et al.* Functional interactors of three genome-wide association study genes are differentially expressed in severe chronic obstructive pulmonary disease lung tissue. *Sci. Rep.* **7,** 44232 (2017).

9. Hobbs, B. D. *et al.* Genetic loci associated with chronic obstructive pulmonary disease overlap with loci for lung function and pulmonary fibrosis. *Nat. Genet.* **49,** 426–432 (2017).

10. Wain, L. V *et al.* Genome-wide association analyses for lung function and chronic obstructive pulmonary disease identify new loci and potential druggable targets. *Nat. Genet.* **49,** 416–425 (2017).

11. Fazekas, D. *et al.* SignaLink 2 – a signaling pathway resource with multi-layered regulatory networks. *BMC Syst. Biol.* **7,** 7 (2013).

12. Hornbeck, P. V *et al.* PhosphoSitePlus: a comprehensive resource for investigating the structure and function of experimentally determined post-translational modifications in man and mouse. *Nucleic Acids Res.* **40,** D261--D270 (2011).

13. Liberzon, A. *et al.* The Molecular Signatures Database Hallmark Gene Set Collection. *Cell Syst.* **1,** 417–425 (2015).

14. Piñero, J. *et al.* DisGeNET: a discovery platform for the dynamical exploration

of human diseases and their genes. *Database (Oxford).* **2015,** bav028 (2015).

15. Torgerson, D. G. *et al.* Meta-analysis of genome-wide association studies of asthma in ethnically diverse North American populations. *Nat. Genet.* **43,** 887–92 (2011).

16. Cho, M. H. *et al.* Risk loci for chronic obstructive pulmonary disease: a genome-wide association study and meta-analysis. *Lancet. Respir. Med.* **2,** 214–25 (2014).

17. Yang, C.-W. *et al.* Regulation of T Cell Receptor Signaling by DENND1B in T H 2 Cells and Allergic Disease. *Cell* **164,** 141–155 (2016).

18. Pelaia, G. *et al.* Mitogen-activated protein kinases and asthma. *J. Cell. Physiol.* **202,** 642–653 (2005).

19. Makinde, T., Murphy, R. F. & Agrawal, D. K. The regulatory role of TGF- β in airway remodeling in asthma. *Immunol. Cell Biol.* **85,** 348–356 (2007).

20. Fedorov, I. A., Wilson, S. J., Davies, D. E. & Holgate, S. T. Epithelial stress and structural remodelling in childhood asthma. *Thorax* **60,** 389–94 (2005).

21. Kwak, H. J. *et al.* The Wnt/β-catenin signaling pathway regulates the development of airway remodeling in patients with asthma. *Exp. Mol. Med.* **47,** e198 (2015).

22. Prakash, Y. *et al.* Neurotrophins in lung health and disease. *Expert Rev. Respir. Med.* **4,** 395–411 (2010).

23. Georas, S. N. & Rezaee, F. Epithelial barrier function: at the front line of asthma immunology and allergic airway inflammation. *J. Allergy Clin. Immunol.* **134,** 509–20 (2014).

24. Vignola, A. M. *et al.* Apoptosis and airway inflammation in asthma. *Apoptosis* **5,** 473–85 (2000).

25. Karimi, K. & Forsythe, P. Natural Killer Cells in Asthma. *Front. Immunol.* **4,** 159 (2013).

26. Guo, R.-B. *et al.* Chronic asthma results in cognitive dysfunction in immature mice. *Exp. Neurol.* **247,** 209–217 (2013).

27. Labor, M. *et al.* Long-term predictors of anxiety and depression in adult patients with asthma. *Wien. Klin. Wochenschr.* **129,** 665–673 (2017).

28. Yeh, J.-J., Lin, C.-L., Hsu, W.-H. & Kao, C.-H. The relationship of depression in asthma–chronic obstructive pulmonary disease overlap syndrome. *PLoS One* **12,** e0188017 (2017).

29. Aisenberg, W. H. *et al.* Defining an olfactory receptor function in airway smooth muscle cells. *Sci. Rep.* **6,** 38231 (2016).

30. Amirian, E. *et al.* Genetic variants in inflammation pathway genes and asthma in glioma susceptibility. *Neuro. Oncol.* **12,** 444–52 (2010).

31. Erle, D. J. & Sheppard, D. The cell biology of asthma. *J. Cell Biol.* **205,** 621–31 (2014).

32. Koopmans, T., Kumawat, K., Halayko, A. J. & Gosens, R. Regulation of actin dynamics by WNT-5A: implications for human airway smooth muscle contraction. *Sci. Rep.* **6,** 30676 (2016).

33. Terzano, C., Romani, S., Paone, G., Conti, V. & Oriolo, F. COPD and Thyroid Dysfunctions. *Lung* **192,** 103–109 (2014).

34. Chung, K. F. Cytokines in chronic obstructive pulmonary disease. *Eur. Respir. J. Suppl.* **34,** 50s–59s (2001).

35. Grundy, S. *et al.* Down Regulation of T Cell Receptor Expression in COPD Pulmonary CD8 Cells. *PLoS One* **8,** e71629 (2013).

36. Hoffmann, J. *et al.* Distinct Differences in Gene Expression Patterns in Pulmonary Arteries of Patients with Chronic Obstructive Pulmonary Disease and Idiopathic Pulmonary Fibrosis with Pulmonary Hypertension. *Am. J. Respir. Crit. Care Med.* **190,** 98–111 (2014).

37. Prakash, Y. S. & Martin, R. J. Brain-derived neurotrophic factor in the airways. *Pharmacol. Ther.* **143,** 74–86 (2014).

38. Barnes, P. J. The cytokine network in asthma and chronic obstructive pulmonary disease. *J. Clin. Invest.* **118,** 3546–56 (2008).

39. Jubrail, J., Kurian, N. & Niedergang, F. Macrophage phagocytosis cracking the defect code in COPD. *Biomed. J.* **40,** 305–312 (2017).

40. Culley, F. J. Natural killer cells in infection and inflammation of the lung. *Immunology* **128,** 151–63 (2009).

41. van den Berge, M. *et al.* Airway gene expression in COPD is dynamic with inhaled corticosteroid treatment and reflects biological pathways associated with disease activity. *Thorax* **69,** 14–23 (2014).

42. Wain, L. V *et al.* Genome-wide association analyses for lung function and chronic obstructive pulmonary disease identify new loci and potential druggable targets. *Nat. Genet.* **49,** 416–425 (2017).

43. Durham, A. L. & Adcock, I. M. The relationship between COPD and lung cancer. *Lung Cancer* **90,** 121–7 (2015).

44. Zhai, R., Yu, X., Shafer, A., Wain, J. C. & Christiani, D. C. The Impact of Coexisting COPD on Survival of Patients With Early-Stage Non-small Cell Lung Cancer Undergoing Surgical Resection. *Chest* **145,** 346–353 (2014).

45. Demedts, I. K., Demoor, T., Bracke, K. R., Joos, G. F. & Brusselle, G. G. Role of apoptosis in the pathogenesis of COPD and pulmonary emphysema. *Respir.*

Res. **7,** 53 (2006).

46. Hosgood, H. D., Menashe, I., He, X., Chanock, S. & Lan, Q. PTEN identified as important risk factor of chronic obstructive pulmonary disease. *Respir. Med.* **103,** 1866–1870 (2009).

47. Lorenowicz, M. J., Fernandez-Borja, M. & Hordijk, P. L. cAMP Signaling in Leukocyte Transendothelial Migration. *Arterioscler. Thromb. Vasc. Biol.* **27,** 1014–1022 (2007).

48. Suzuki, M. *et al.* Decreased Airway Expression of Vascular Endothelial Growth Factor in Cigarette Smoke-Induced Emphysema in Mice and COPD Patients. *Inhal. Toxicol.* **20,** 349–359 (2008).

49. Houssaini, A. *et al.* Targeting the mTOR signaling pathway to inhibit lung cell senescence in COPD. in *3.3 Mechanisms of Lung Injury and Repair* **48,** PA4024 (European Respiratory Society, 2016).

50. Houssaini, A. *et al.* mTOR pathway activation drives lung cell senescence and emphysema. *JCI Insight* **3,** (2018).

51. Vallath, S., Hynds, R. E., Succony, L., Janes, S. M. & Giangreco, A. Targeting EGFR signalling in chronic lung disease: therapeutic challenges and opportunities. *Eur. Respir. J.* **44,** 513–22 (2014).

52. Litsiou, E. *et al.* CXCL13 Production in B Cells via Toll-like Receptor/Lymphotoxin Receptor Signaling Is Involved in Lymphoid Neogenesis in Chronic Obstructive Pulmonary Disease. doi:10.1164/rccm.201208-1543OC

53. Mizuno, S. *et al.* p53 Signaling Pathway Polymorphisms Associated With Emphysematous Changes in Patients With COPD. *Chest* **152,** 58–69 (2017).

54. Lee, K.-Y. *et al.* Proteasome activity related with the daily physical activity of

COPD patients. *Int. J. Chron. Obstruct. Pulmon. Dis.* **Volume 12,** 1519–1525 (2017).

55. Caramori, G. *et al.* Unbalanced oxidant-induced DNA damage and repair in COPD: a link towards lung cancer. *Thorax* **66,** 521–7 (2011).

Supplementary Figure and Table Legends

Supplementary Figure 1: The difference in network size and the degree distributions of GRN and EGRN.

a. The in- and out-degree distributions of GRN and EGRN.

b. Normalized control centrality (c_c=C_c/N) distributions of GRN and EGRN.

Supplementary Figure 2: The control centrality measure of T2D pathway genes compared to random distribution.

The distribution of control centralities for T2D pathway genes versus that of the same number of random genes in EGRN.

Supplementary Figure 3: Comparison of HiCc pathways with other methods.

a. The overlap of HiCc pathways with T2D-related pathways (shown with the arrow) and the overlap of the 66 JActive modules with true positive pathways (blue bars), measured by the Jaccard index.

b. The overlap of HiCc pathways with T2D-related pathways (shown with the arrow) and the overlap of the 66 JActive modules with true positive pathways (blue bars), measured by the Fisher's exact test p-value.

c. Statistics of the largest subnetworks of HotNet2 for various delta values including their T2D-related pathway enrichments.

Supplementary Figure 4: Properties and disease relevance of asthma high control centrality (HiCc) pathways.

a. Degree distributions P(k) of HiCc pathway genes, non-HiCc pathway genes, and all other genes in the asthma EGRN.

b. The fraction of enriched pathways in the asthma GOLD and GWAS datasets, for HiCc pathways, non-HiCc pathways, and all pathways.

c. The 32 asthma HiCc pathways and their enrichment in asthma-specific data sources.

Supplementary Figure 5: Properties and disease relevance of COPD high control centrality (HiCc) pathways.

a. Degree distributions P(k) of HiCc pathway genes, non-HiCc pathway genes, and all other genes in the COPD EGRN.

b. The fraction of enriched pathways in the COPD GOLD and GWAS datasets, for HiCc pathways, non-HiCc pathways, and all pathways.

c. The 42 COPD HiCc pathways and their enrichment in COPD-specific data sources.

Supplementary Figure 6: Summary of the HiCc pathway identification approach.

NFATC4 gene with a cis-eQTL (rs79584546) was found to be involved in four HiCc pathways.

Supplementary Figure 7: NFATC4 silencing *in vitro*.

The silenced NFATC4 mRNA levels under four different conditions.

Supplementary Figure 8: Correlation of NFATC4 expression with putative downstream genes in islet data.

Spearman correlation of NFATC4 expression in islet cells with putative downstream genes. See Supplementary Table 6 for correlation and p-values.

Supplementary Table 1: Comparison of control centrality with other centrality measures.

The T2D-related pathway enrichment of high control centrality pathways, compared to other high-centrality pathways.

Supplementary Table 2: The HiCc pathways' enrichment in T2D omics data.

Arranged based on the significance in T2D specific -omics data.

Supplementary Table 3: Characteristics of Pancreatic donors in Nordic Islet Transplantation Programme.

Supplementary Table 4: The list of curated GWAS genes.

Supplementary Table 5: Probes of putative downstream genes of NFATC4.

Supplementary Table 6: NFATC4 expression in islet cells was found to be significantly positively correlated with nine putative downstream genes and significantly negatively correlated with one putative downstream gene.

Supplementary Figure 1

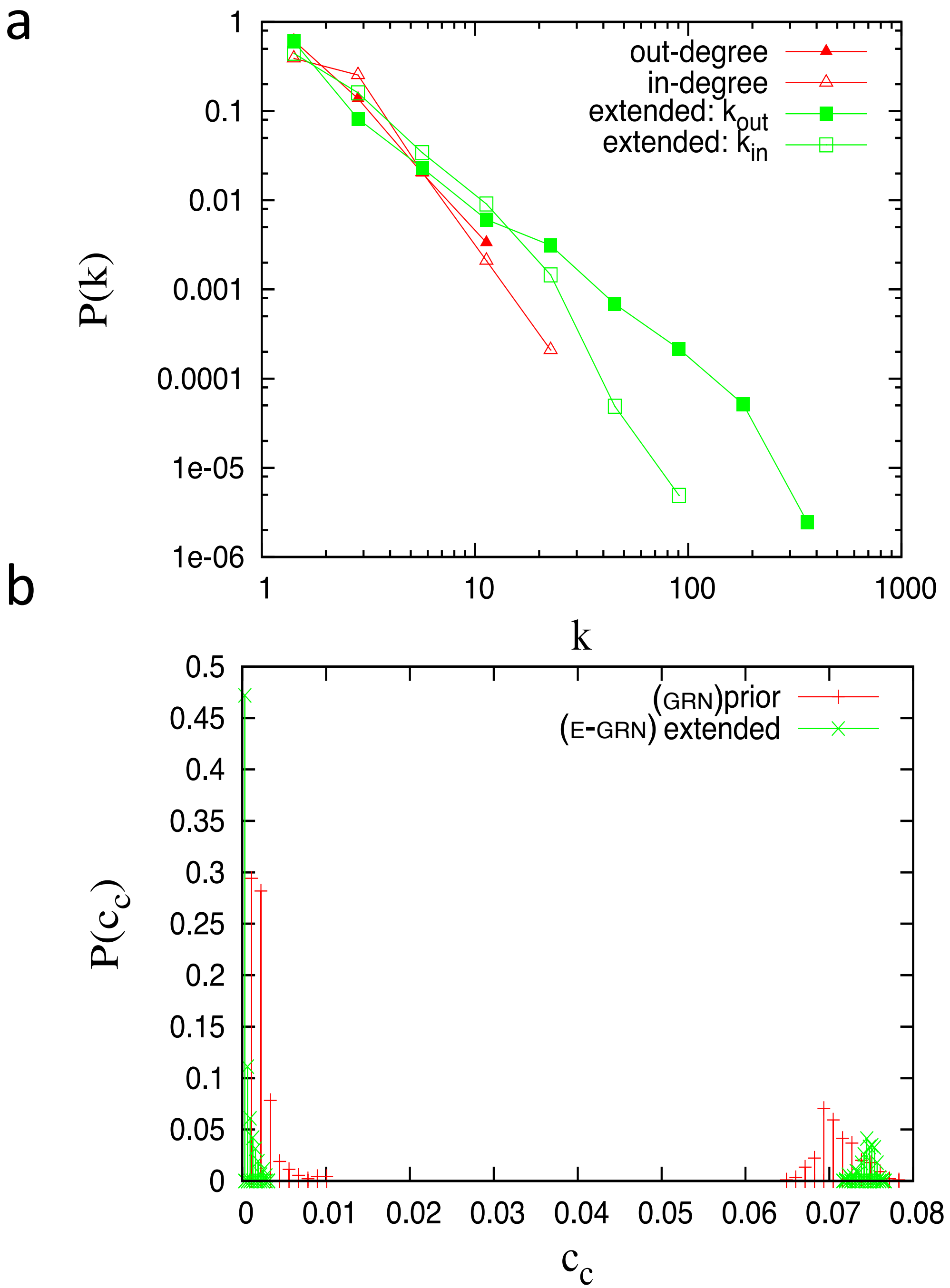

Supplementary Figure 2

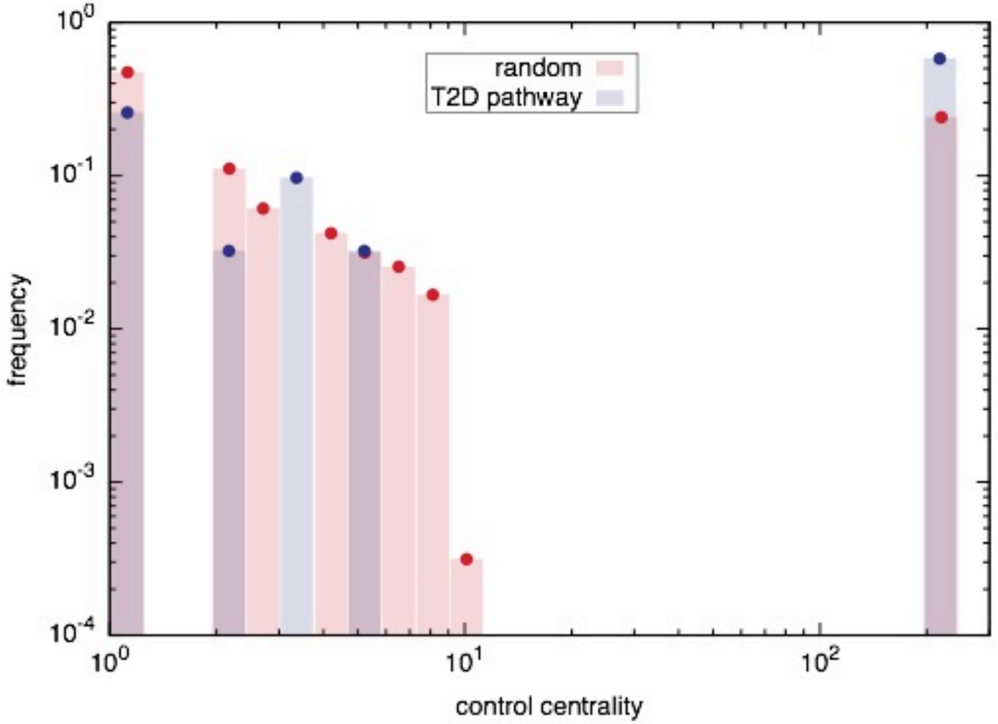

Supplementary Figure 3

a

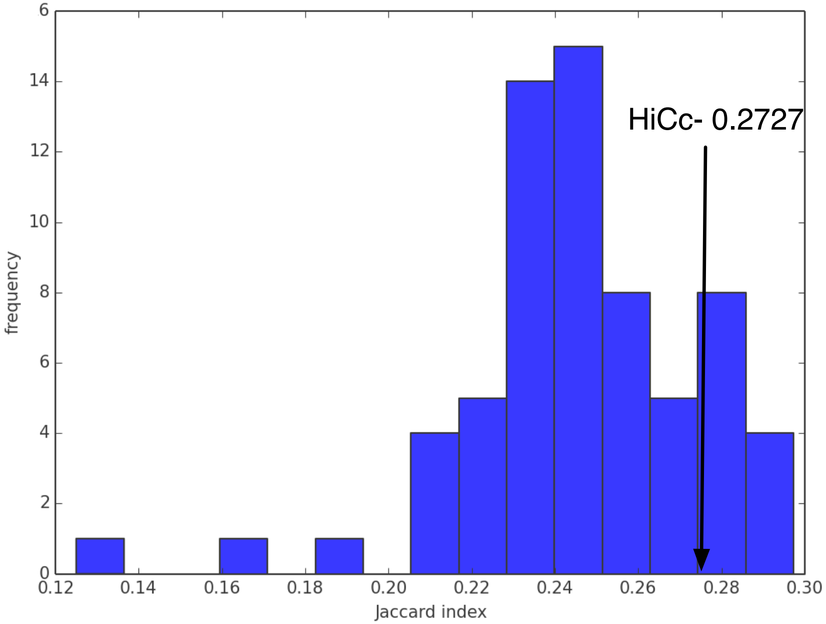

b

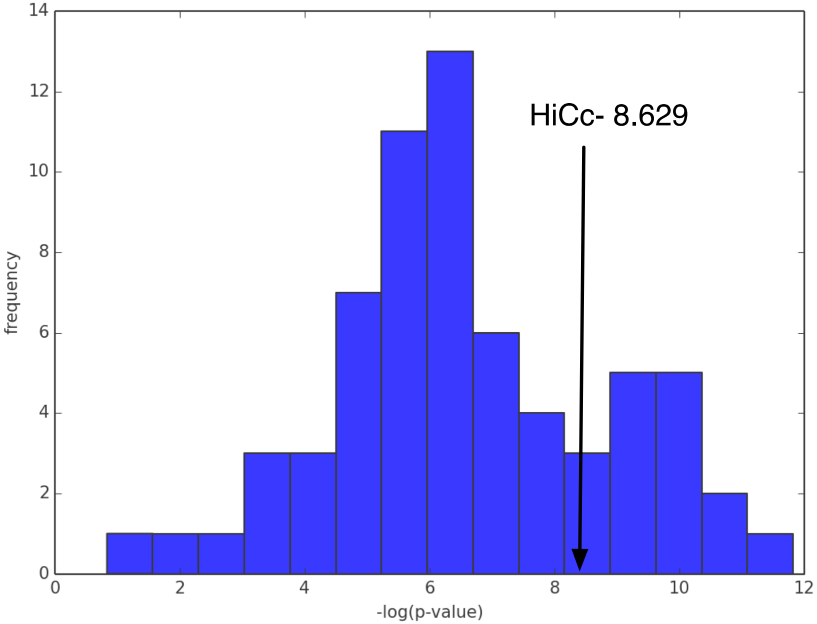

c

	Largest subnetwork size	# significant (KEGG) pathways	# significant T2D related pathways	P-value (Fisher's Exact test)	-log P	Jaccard
Delta=0.026	39	33	13	0.000624	7.3799	0.25
Delta=0.029	32	25	9	0.018403	3.9952	0.18
Delta=0.036	16	15	2	1.0	0	0.04
Delta=0.063	9	0	0	NA	NA	NA

Supplementary Figure 4

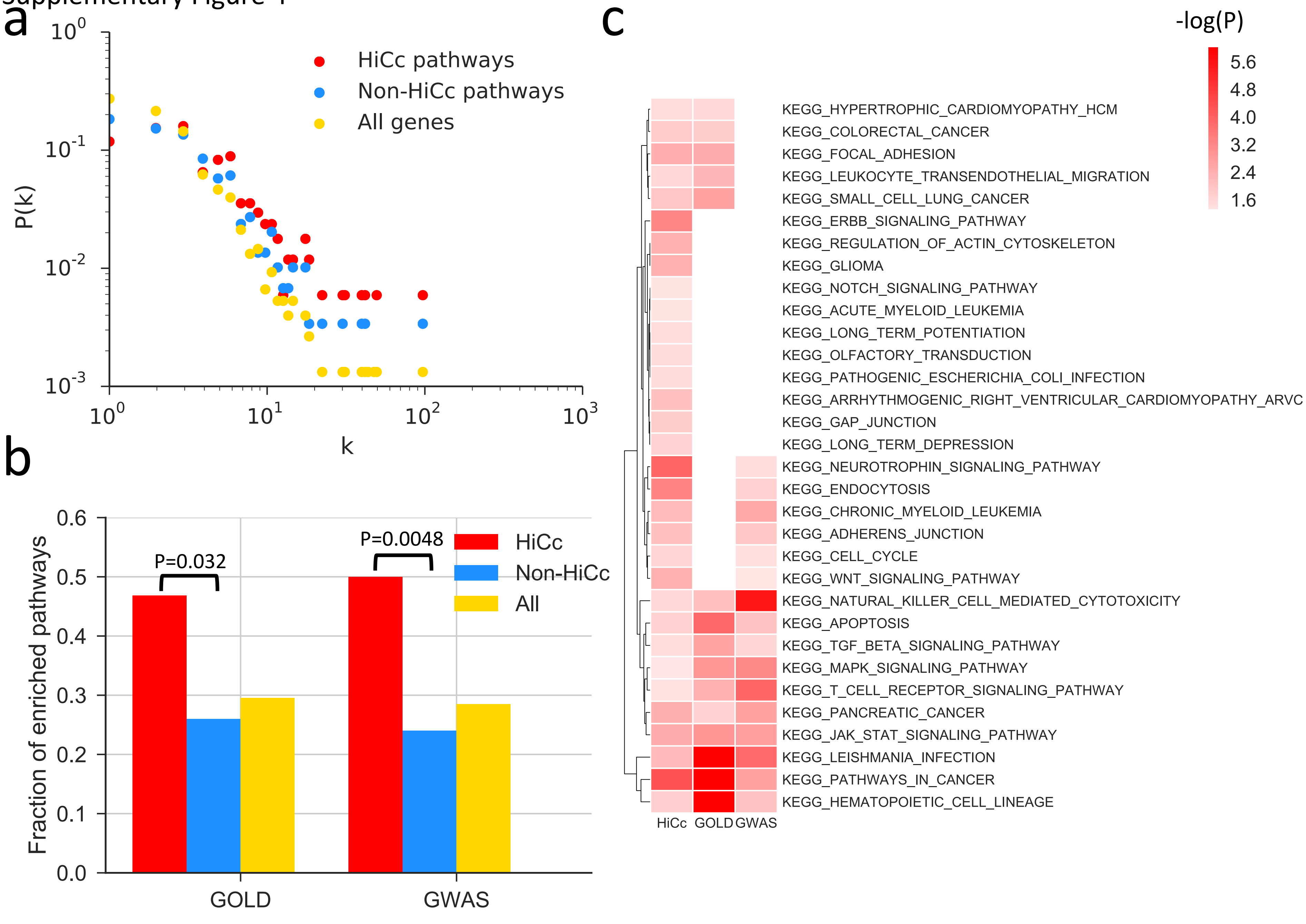

Supplementary Figure 5

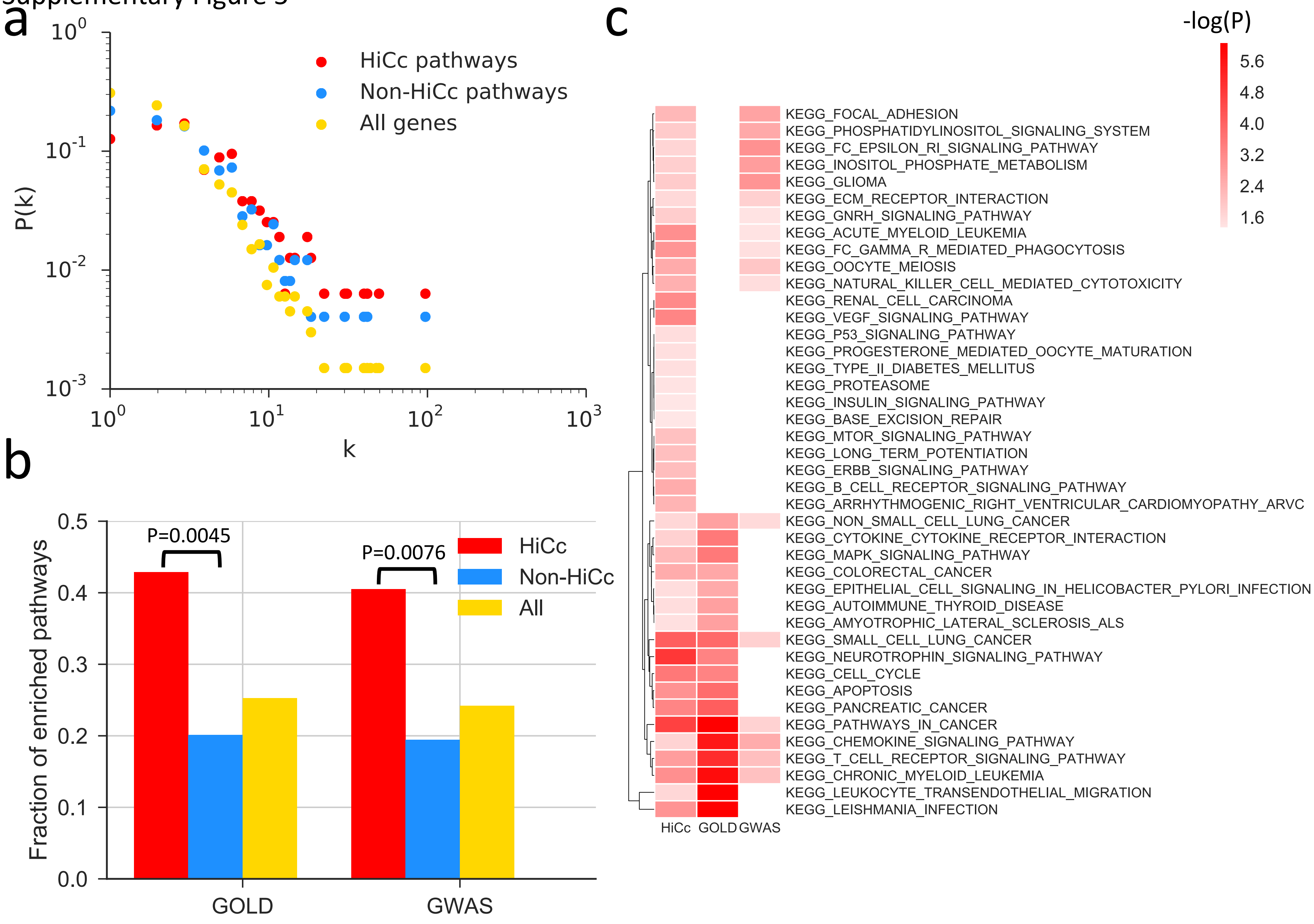

Supplementary Figure 6

Extended-Gene-regulatory network
N=3,084 genes and M=7,935 edges, with an average shortest path length <l> = 4.65

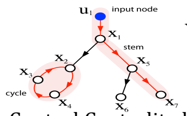

Control Centrality based pathway ranking in T2D

KEGG pathways
N=186

Significant pathways with high control centrality genes
66 pathways in total

eQTL analysis of significant pathway-genes
In total, we observed a SNP within 250 up or downstream of the genes in 33 pathways.

Functional-network and 51 eQTLs in 33 pathways
The pairs of eQTLs genes with shortest path of one and two was significantly greater (17.3%) compared to all gene (4.8%) pairs in the network (p-0.01).

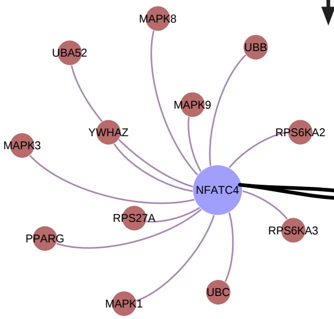

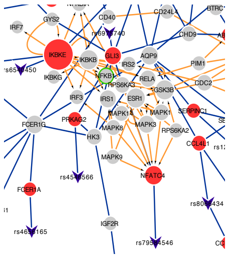

cis-eQTL
rs79584546 -FDR-1.78E-04

NFATC4 is part of:
Wnt signaling pathway
B-cell Receptor signaling
MAPK signaling
T-cell Receptor signaling

Supplementary Figure 7

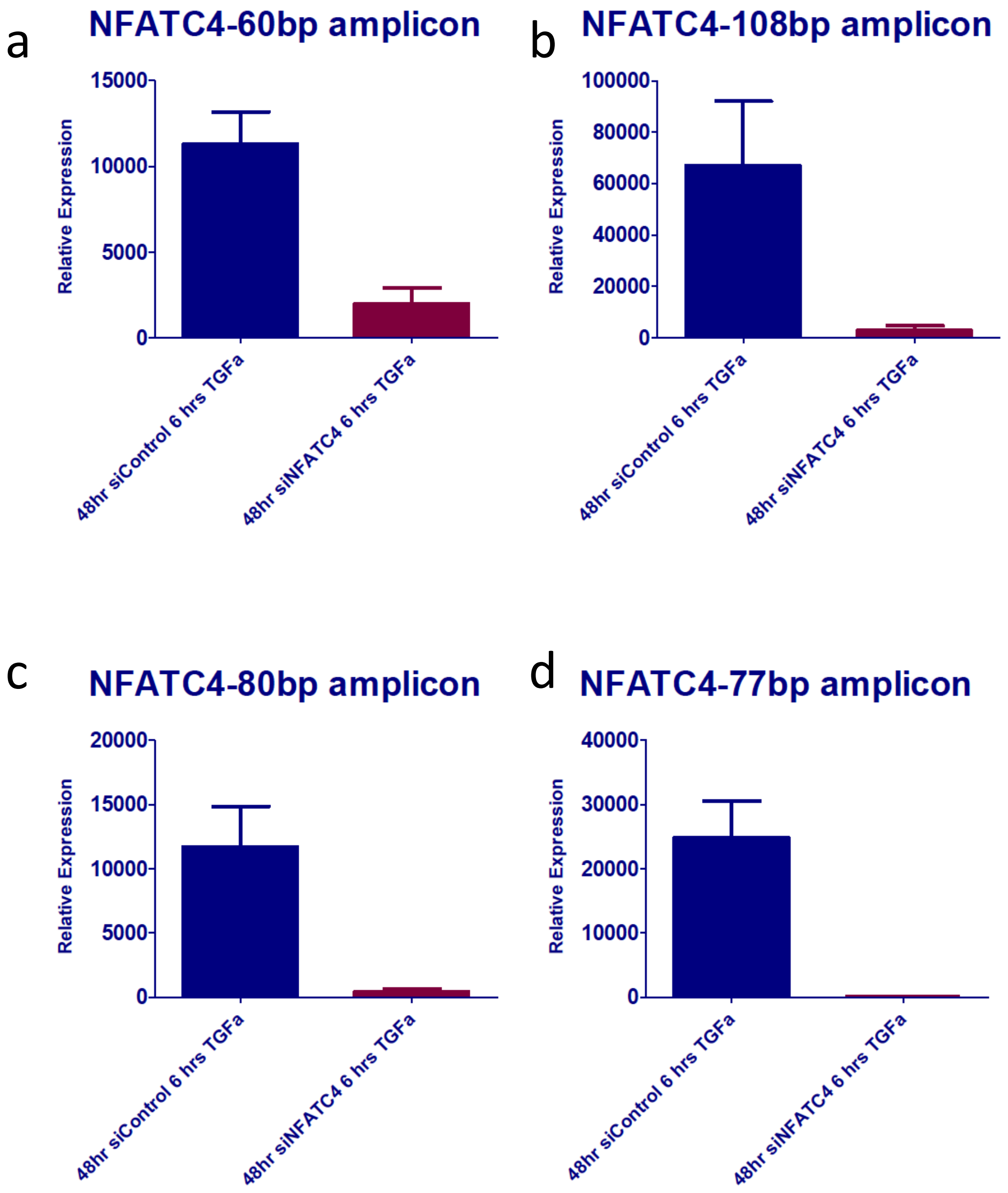

Supplementary Figure 8

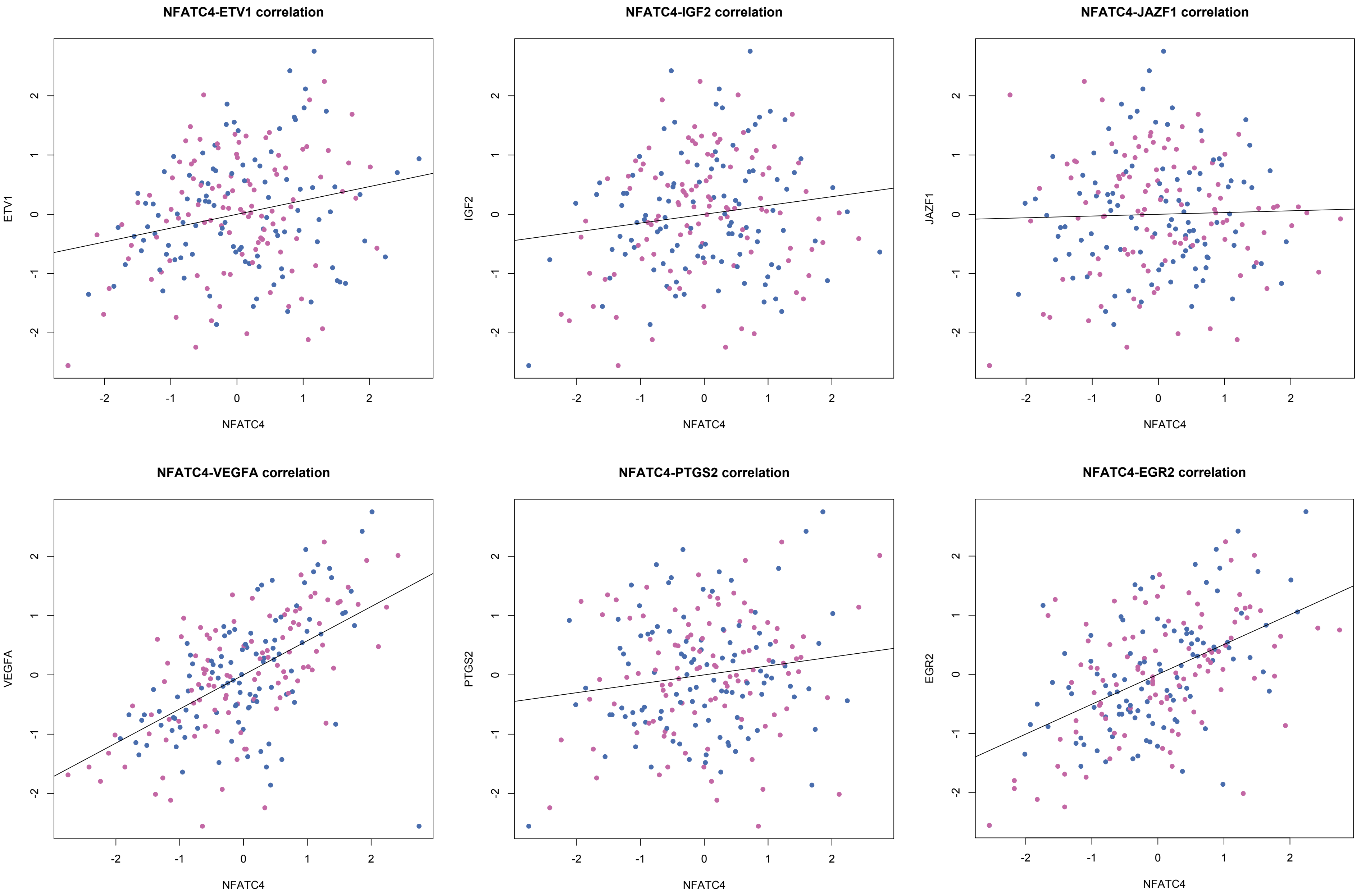

Supplementary Figure 8 (cont'd)

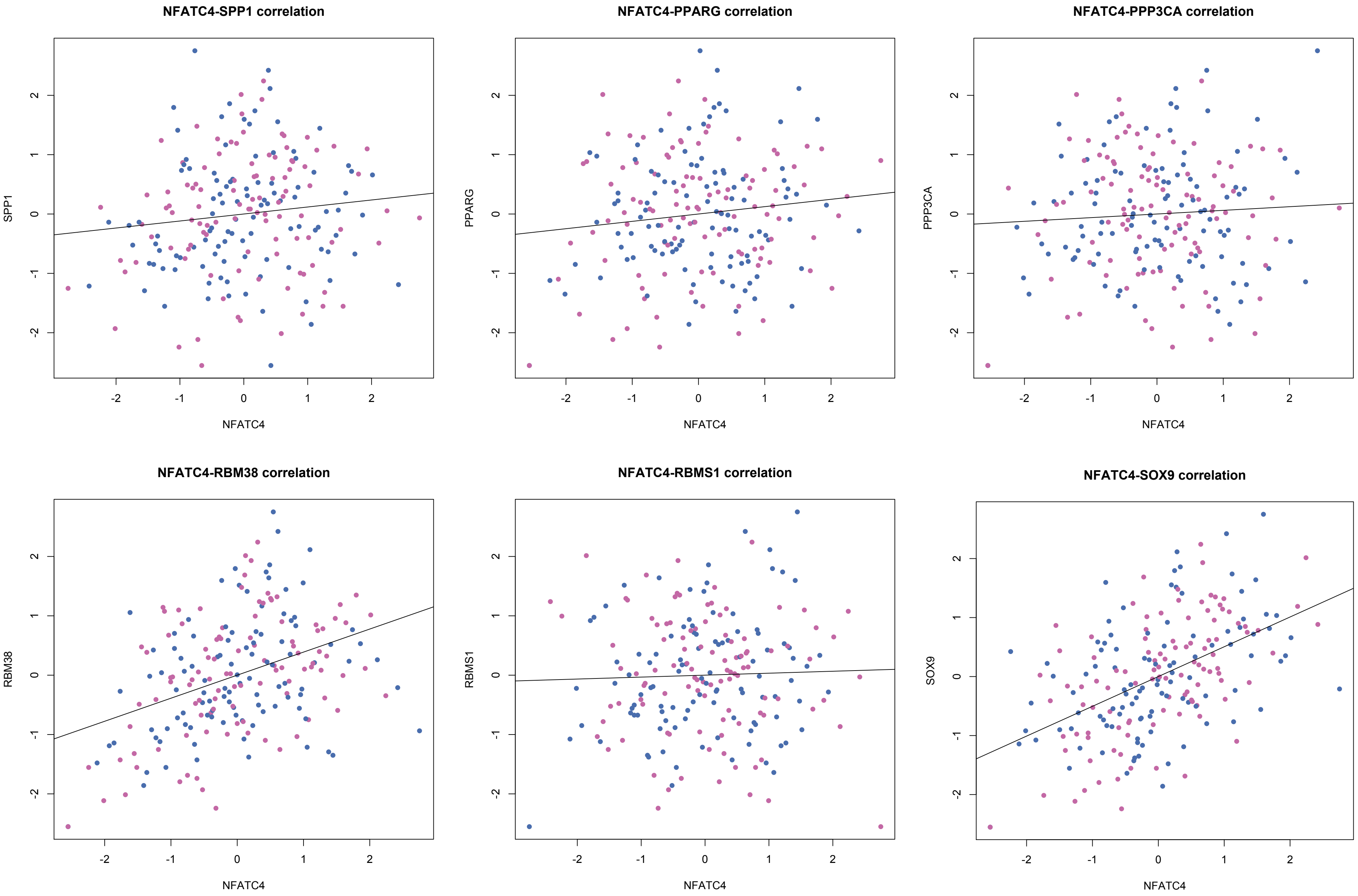

Supplementary Figure 8 (cont'd)

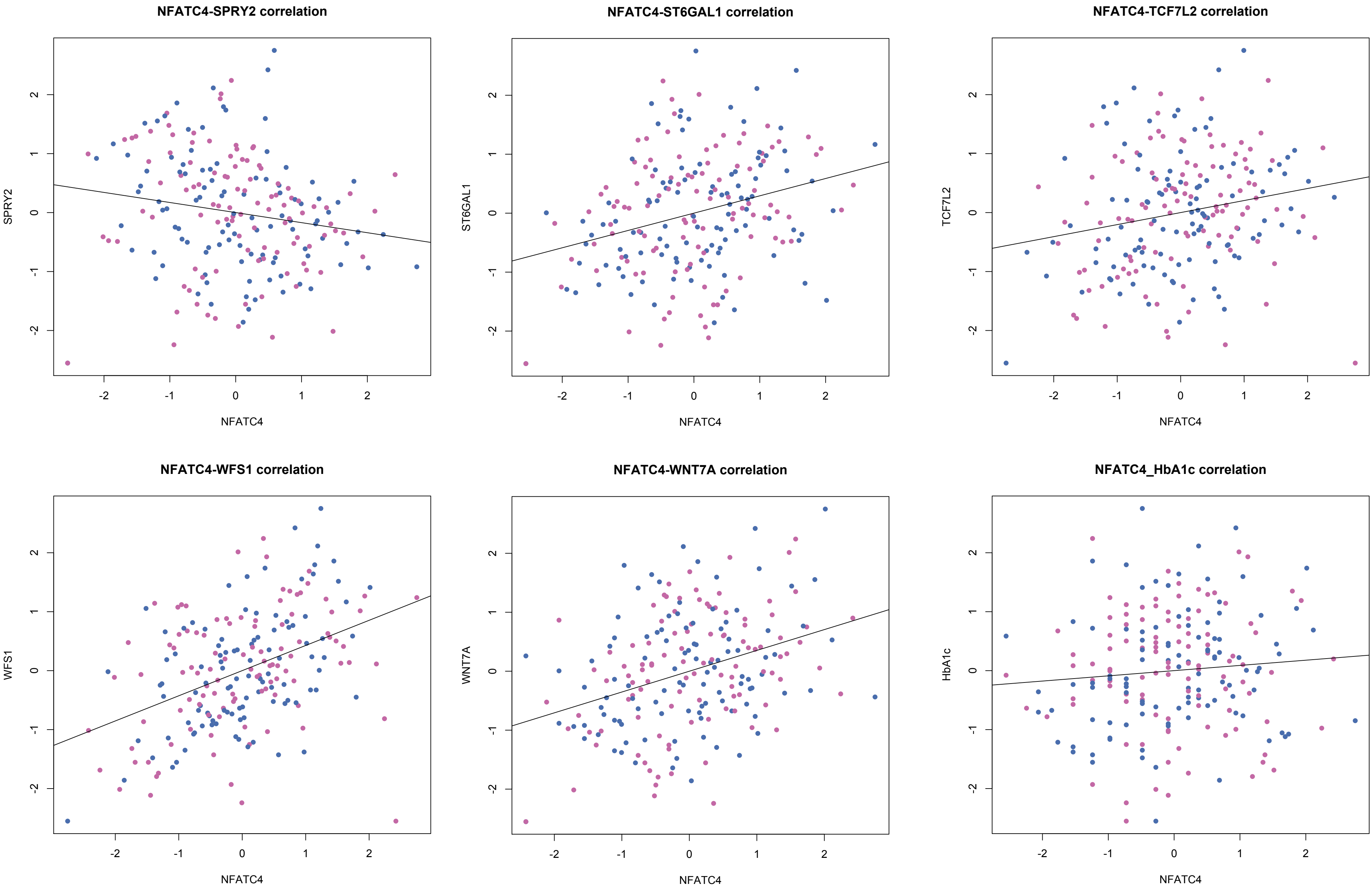

Supplementary Table 1

Centrality measure	T2D-related/ all high centrality pathways	P-value (Fisher's Exact test)	-log P	Jaccard index
Control	21/66	0.00018	8.63	0.27
Betweenness	23/82	0.00072	7.23	0.25
Degree	20/74	0.00525	5.24	0.23
PageRank	20/76	0.0095	4.66	0.23
Eigenvector	17/69	0.0457	3.08	0.20
Closeness	17/74	0.112	2.18	0.19

Supplementary Table 2

Pathway	HiCc_p-value	GOLD_p-value	GWAS_p-value
KEGG_TYPE_II_DIABETES_MELLITUS	5.76E-05	7.43E-20	2.71E-04
KEGG_PPAR_SIGNALING_PATHWAY	3.17E-04	9.33E-16	1.93E-02
KEGG_INSULIN_SIGNALING_PATHWAY	9.01E-03	2.85E-16	1.51E-02
KEGG_CALCIUM_SIGNALING_PATHWAY	1.05E-04	1.97E-03	4.28E-10
KEGG_PATHWAYS_IN_CANCER	4.50E-05	5.85E-06	4.42E-06
KEGG_CYTOKINE_CYTOKINE_RECEPTOR_INTERACTION	3.95E-07	7.80E-06	1.35E-03
KEGG_CHEMOKINE_SIGNALING_PATHWAY	7.72E-08	1.49E-02	1.91E-04
KEGG_BLADDER_CANCER	2.56E-08	1.84E-02	2.65E-03
KEGG_HEMATOPOIETIC_CELL_LINEAGE	1.65E-06	9.74E-04	1.12E-03
KEGG_GLIOMA	1.86E-08	4.14E-02	4.67E-03
KEGG_MELANOMA	5.90E-09	4.85E-02	2.49E-02
KEGG_ENDOCYTOSIS	3.32E-02	2.22E-03	7.13E-06
KEGG_PROSTATE_CANCER	1.83E-04	1.02E-03	6.62E-03
KEGG_ADHERENS_JUNCTION	5.17E-03	5.33E-04	1.76E-03
KEGG_PANCREATIC_CANCER	1.30E-03	4.73E-02	1.02E-02
KEGG_ABC_TRANSPORTERS	8.27E-03	2.01E-02	1.15E-02
KEGG_P53_SIGNALING_PATHWAY	2.37E-02	4.61E-02	3.92E-02
KEGG_ADIPOCYTOKINE_SIGNALING_PATHWAY	9.52E-04	2.15E-19	1.00E+00
KEGG_GNRH_SIGNALING_PATHWAY	3.15E-16	1.00E+00	1.17E-04
KEGG_MAPK_SIGNALING_PATHWAY	4.27E-12	1.00E+00	4.85E-06
KEGG_VASCULAR_SMOOTH_MUSCLE_CONTRACTION	9.21E-09	1.00E+00	6.10E-08
KEGG_FOCAL_ADHESION	1.67E-06	1.00E+00	3.69E-10
KEGG_FC_EPSILON_RI_SIGNALING_PATHWAY	7.39E-09	1.00E+00	5.64E-04
KEGG_PROGESTERONE_MEDIATED_OOCYTE_MATURATION	4.17E-10	1.00E+00	3.62E-02
KEGG_GAP_JUNCTION	1.04E-07	1.00E+00	2.57E-04
KEGG_ECM_RECEPTOR_INTERACTION	8.88E-03	1.00E+00	3.45E-09
KEGG_LONG_TERM_DEPRESSION	8.30E-04	1.00E+00	5.66E-07
KEGG_MELANOGENESIS	6.53E-08	1.00E+00	8.25E-03
KEGG_COMPLEMENT_AND_COAGULATION_CASCADES	7.10E-07	1.00E+00	1.51E-03
KEGG_T_CELL_RECEPTOR_SIGNALING_PATHWAY	7.27E-06	1.00E+00	3.98E-04
KEGG_ERBB_SIGNALING_PATHWAY	1.20E-08	1.00E+00	1.00E+00
KEGG_ENDOMETRIAL_CANCER	4.88E-07	2.75E-02	1.00E+00
KEGG_TOLL_LIKE_RECEPTOR_SIGNALING_PATHWAY	1.11E-06	1.43E-02	1.00E+00

Supplementary Table 2 (continued)

Pathway	HiCc p-value	GOLD p-value	GWAS p-value
KEGG_WNT_SIGNALING_PATHWAY	5.00E-04	1.00E+00	3.59E-05
KEGG_DILATED_CARDIOMYOPATHY	7.49E-03	1.00E+00	1.86E-05
KEGG_PRION_DISEASES	1.20E-04	1.00E+00	1.44E-03
KEGG_LONG_TERM_POTENTIATION	1.11E-04	1.00E+00	1.81E-03
KEGG_OLFACTORY_TRANSDUCTION	2.75E-07	1.00E+00	1.00E+00
KEGG_NOD_LIKE_RECEPTOR_SIGNALING_PATHWAY	8.62E-05	3.63E-03	1.00E+00
KEGG_MTOR_SIGNALING_PATHWAY	2.64E-03	1.30E-04	1.00E+00
KEGG_FATTY_ACID_METABOLISM	1.57E-03	1.00E+00	2.29E-04
KEGG_TIGHT_JUNCTION	1.43E-02	1.00E+00	2.86E-05
KEGG_NON_SMALL_CELL_LUNG_CANCER	4.70E-05	1.00E+00	1.06E-02
KEGG_SMALL_CELL_LUNG_CANCER	6.94E-03	1.00E+00	2.12E-04
KEGG_HEDGEHOG_SIGNALING_PATHWAY	1.92E-04	1.00E+00	1.46E-02
KEGG_OOCYTE_MEIOSIS	6.34E-04	1.00E+00	4.44E-03
KEGG_COLORECTAL_CANCER	4.45E-06	1.00E+00	1.00E+00
KEGG_NEUROTROPHIN_SIGNALING_PATHWAY	1.19E-04	1.00E+00	4.86E-02
KEGG_THYROID_CANCER	7.45E-04	9.06E-03	1.00E+00
KEGG_PROPANOATE_METABOLISM	3.66E-02	1.00E+00	1.92E-04
KEGG_VEGF_SIGNALING_PATHWAY	1.25E-05	1.00E+00	1.00E+00
KEGG_TGF_BETA_SIGNALING_PATHWAY	5.53E-03	1.00E+00	4.35E-03
KEGG_REGULATION_OF_ACTIN_CYTOSKELETON	1.38E-02	1.00E+00	1.76E-03
KEGG_LYSOSOME	9.57E-03	1.00E+00	5.19E-03
KEGG_ACUTE_MYELOID_LEUKEMIA	1.52E-03	3.58E-02	1.00E+00
KEGG_EPITHELIAL_CELL_SIGNALING_IN_HELICOBACTER_PYLORI_INFECTION	5.88E-05	1.00E+00	1.00E+00
KEGG_VIBRIO_CHOLERAE_INFECTION	7.56E-03	1.00E+00	1.46E-02
KEGG_ALPHA_LINOLENIC_ACID_METABOLISM	1.28E-02	1.00E+00	9.15E-03
KEGG_NATURAL_KILLER_CELL_MEDIATED_CYTOTOXICITY	4.02E-02	1.00E+00	8.38E-03
KEGG_TASTE_TRANSDUCTION	4.10E-04	1.00E+00	1.00E+00
KEGG_B_CELL_RECEPTOR_SIGNALING_PATHWAY	3.69E-02	1.00E+00	2.01E-02
KEGG_APOPTOSIS	1.39E-03	1.00E+00	1.00E+00
KEGG_ARACHIDONIC_ACID_METABOLISM	1.38E-02	1.00E+00	1.00E+00
KEGG_RIG_I_LIKE_RECEPTOR_SIGNALING_PATHWAY	1.78E-02	1.00E+00	1.00E+00
KEGG_CHRONIC_MYELOID_LEUKEMIA	2.53E-02	1.00E+00	1.00E+00
KEGG_BASAL_CELL_CARCINOMA	3.77E-02	1.00E+00	1.00E+00

Supplementary Table 3

Characteristics of human pancreatic donors.

	Non-diabetic donors	T2D donors	p-value
n (male/female)	55 (29/26)	9(5/4)	
Age (years)	56.7 ± 9.8	57.0 ± 13.1	0.99
BMI (kg/m2)	25.9 ± 3.6	28.5 ± 4.7	0.13
HbA1c (%)	5.7 ± 0.8	7.3 ± 1.2	0.0001
HbA1c (mmol/mol)	38.8 ± 8.7	56.2 ± 12.9	0.0001
Basal insulin secretion (ng/islet/h)	0.22 (0.39)	0.22 (0.17)	0.87
Glucose-stimulated insulin secretion (ng/islet/h)	1.06 (0.86)	0.54 (0.56)	0.025
Insulin content (ng/ml)	18650 (16881)	9370 (9244)	0.043

Supplementary Table 4

Gene/Nearest Gene	T2D Risk SNP	Gene Location	Chr	RA	OA	OR	TRAIT	Reference
DGKB/TMEM195	rs2191349	intergenic	7	T	G	1.06	Fasting glucose/T2D	PMID: 20081858
PROX1	rs340874	intergenic	1	C	T	1.07	Fasting glucose/T2D	PMID: 20081858
CRY2	rs11605924	intron	11	A	C	1.04	Fasting glucose/T2D	PMID: 20081858
G6PC2/ABCB11	rs560887	intron	2	T	C	1.03	Fasting glucose/T2D	PMID: 20081858
FAM148B	rs11071657	intergenic	3	A	G	1.03	Fasting glucose/T2D	PMID: 20081858
GCK	rs4607517	intergenic	7	A	G	1.07	Fasting glucose/T2D	PMID: 20081858
FADS1	rs174550	intron	11	T	C	1.04	Fasting glucose/T2D	PMID: 20081858
IRS1	rs2943641	intergenic	2	C	T	1.19	Fasting glucose/T2D	PMID: 19734900
IGF1	rs35767	nearGene-5	12	G	A	1.04	Fasting insulin/T2D/	PMID: 20081858
KLF14	rs972283	intergenic	7	G	A	1.07	Reduced insulin sensitivity T2D	PMID: 20581827
NOTCH2	rs10923931	intron	1	T	G	1.13	T2D	PMID: 19020324, 18372903
BCL11A	rs243021	intergenic	2	A	G	1.08	T2D	PMID: 20581827
THADA	rs7578597	coding-missense	2	T	C	1.15	T2D	PMID: 19020324, 18372903
ADAMTS9-AS2	rs4607103	intron	3	C	T	1.09	T2D	PMID: 19020324, 18372903
IGF2BP2	rs4402960	intron	3	T	G	1.11	T2D	PMID: 17463246
PPARG	rs1801282	coding-missense	3	C	G	1.09	T2D	PMID: 17463246
WFS1	rs10010131	intron	4	G	A	1.14	T2D	PMID: 19020324, 17603484
ZBED3	rs4457053	intron of ZBED3-AS1	5	G	A	1.08	T2D	PMID: 20581827
CDKAL1	rs7754840	intron	6	C	G	1.17	T2D	PMID: 20581827
JAZF1	rs864745	intron	7	T	C	1.1	T2D	PMID: 19020324, 18372903
SLC30A8	rs13266634	coding-missense	8	C	T	1.19	T2D	PMID: 17293876
TP53INP1	rs896854	intron	8	T	C	1.06	T2D	PMID: 20581827
CDKN2A/2B	rs10965250	intergenic	9	G	A	1.2	T2D	PMID: 20581827,17463246
CDKN2B	rs10811661	intergenic	9	T	C	1.2	T2D	PMID: 17463246, 20581827
GLIS3	rs7041847	intron	9	A	G	1.1	T2D	PMID: 22158537,22961080
TLE4 (CHCHD9)	rs13292136	intergenic	9	C	T	1.11	T2D	PMID: 20581827
ADRA2A	rs553668	UTR-3	10	A	G	1.42	T2D	PMID: 19965390
CDC123, CAMK1D	rs12779790	intergenic	10	G	A	1.11	T2D	PMID: 19020324, 18372903
HHEX	rs5015480	intergenic	10	C	T	1.13	T2D	PMID: 20581827,17463246
TCF7L2	rs7901695	intron	10	C	T	1.37	T2D	PMID: 20581827,17463246
KCNJ11	rs5219	coding-missense	11	T	C	1.14	T2D	PMID: 17463248
KCNQ1	rs2237895	intron	11	C	T	1.45	T2D	PMID: 18711367
MTNR1B	rs10830963	intron	11	G	C	1.09	T2D	PMID: 19060907
HMGA2	rs1531343	intron of pseudogene	12	C	G	1.1	T2D	PMID: 20581827
HNF1A	rs7305618	intergenic	12	C	T	1.14	T2D	PMID: 20581827,21573907
TSPAN8, LGR5	rs7961581	intergenic	12	C	T	1.09	T2D	PMID: 19020324, 18372903
PRC1	rs8042680	intron	15	A	C	1.07	T2D	PMID: 20581827
ZFAND6	rs11634397	intergenic	15	G	A	1.06	T2D	PMID: 20581827
FTO	rs11642841	intron	16	A	C	1.13	T2D	PMID: 20581827,17463248
HNF1B	rs7501939	intron	17	T	C	1.09	T2D	PMID: 17603485
GIPR	rs8108269	intergenic	19	G	T	1.05	T2D	PMID: 22885922
DUSP9	rs5945326	intergenic	X	A	G	1.27	T2D	PMID: 20581827
MADD	rs10501320	intron	11	G	C	1.01	T2D fasting proinsulin levels/fasting glucose	PMID: 21873549
ARAP1	rs11603334	UTR-5	11	G	A	1.13	T2D fasting proinsulin levels/fasting glucose/	PMID: 21873549
ADCY5	rs11708067	intron	3	A	G	1.12	T2D/2hr glucose/	PMID: 20081858
GCKR	rs780094	intron	2	C	T	1.06	T2D/Fasting glucose	PMID: 20081858
VPS13C/C2CD4A/B	rs4502156	intergenic	15	T	C	1.07	fasting proinsulin levels T2D	PMID: 21873549
SLC2A2	rs11920090	intron	3	T	A	NA	Fasting glucose	PMID: 20081858

Supplementary Table 5

Gene	Name	Species	Ref#
Pex5l	Peroxisomal biogenesis factor 5 like	Rat	Rn00597720_m1
Rbms1	RNA binding motif single stranded interacting protein 1	Rat	Rn01513258_m1
Rbm38	RNA binding motif protein 38	Rat	Rn01528881_g1
ARL15	ADP ribosylation factor like GTPase 15	Rat	Rn01494252_m1
SPRY2	Sprouty RTK signaling antagonist 2	Rat	Rn02534289_s1
WFS1	Wolframin ER transmembrane glycoprotein	Rat	Rn00582735_m1
ETV1	ETS (E twenty-six) variant 1	Rat	Rn01472807_m1
JAZF1	JAZF zinc finger 1	Rat	Rn01409910_m1
ST6GAL1	ST6 beta-galactoside alpha-2,6-sialyltransferase 1	Rat	Rn00709937_m1
VEGF-A	Vascular endothelial growth factor A	Rat	Rn01511602_m1
SLC44A3	Solute carrier family 44 member 3	Rat	Rn01523535_m1
WNT7A	Wnt family member 7A	Rat	Rn01425352_m1
SOX9	SRY-box 9	Rat	Rn01751070_m1
Ppp3ca	Calcineurin A	Mouse	Mm01317678_m1
IGF-2	Insulin-like growth factor 2	Rat	Rn01454518_m1
EGR2	Early growth response 2	Rat	Rn00586224_m1
COX-2	Cyclooxygenase-2; PTGS-2	Rat	Rn01483828_m1
NFATC4	NFATC4 60 bp amplicon	Rat	Rn01488725_m1
NFATC4	NFATC4 106 bp amplicon	Rat	Rn01488727_m1
NFATC4	NFATC4 80 bp amplicon	Rat	Rn01488724_m1
NFATC4	NFATC4 77 bp	Rat	Rn01488729_m1
OPN	Osteopontin (OPN) or bone sialoprotein (BSP-1)	Rat	Rn00681031_m1
PPAR-γ	Peroxisome proliferator-activated receptor gamma	Rat	Rn00440945_m1
TCF7L2	Transcription factor 7 like 2	Rat	Rn01411019_m1
GAPDH	Endogenous control	Rat	Rn01775763_g1
RPLP0	Endogenous control	Rat	Rn003342271_gH

Supplementary Table 6

NFATC4	rho	p-value	FDR
ETV1	0.1758074	0.01069846	0.01818738
IGF2	0.1170383	0.09069521	0.1284849
JAZF1	-0.006345213	0.9271724	0.9851207
VEGFA	0.5849896	1.12779e-20	1.917243e-19
ARF15			
PTGS2/COX2	0.1066532	0.1233838	0.1613480
EGR2	0.4778676	2.226549e-13	1.261711e-12
SPP1 (OPN)	0.1320623	0.05604067	0.0866083
PEX5I			
PPARG	0.06182413	0.3727027	0.4525676
PPP3CA	-0.0002662867	0.9969395	0.9969395
RBM38	0.3694562	3.419713e-08	1.162702e-07
RBMS1	0.0348829	0.6152194	0.6972487
SIC44A3			
SOX9	0.5068847	4.155743e-15	3.532382e-14
SPRY2	-0.2446196	0.0003459254	0.0007350915
ST6GAL1	0.2785855	4.233712e-05	0.0001028187
TCF7L2	0.2275008	0.0008976801	0.001695618
WFS1	0.4215794	1.861908e-10	7.913109e-10
WNT7A	0.3245769	1.537845e-06	4.357228e-06
Correlation with HbA1c		0.1558124	
	pval	0.03227819	
